# Supplementary material for: Disruption of the grid cell network in a mouse model of early Alzheimer’s disease
Source: Nat Commun. 2022 Feb 16;13:886. doi: 10.1038/s41467-022-28551-x (PMC8850598; doi:10.1038/s41467-022-28551-x)
Supplement: Supplementary file 1 — Supplementary Information [file 41467_2022_28551_MOESM1_ESM.pdf]

Supplementary Information for

Disruption of the grid cell network in a mouse model of early Alzheimer's disease

Johnson Ying, Alexandra T. Keinath, Raphael Lavoie, Erika Vigneault, Salah El Mestikawy, Mark P. Brandon

*Correspondence to: mark.brandon@mcgill.ca

Contains:

- **Supplementary Tables 1-2**
- **Supplementary Figures 1-24**

Supplementary Table 1. Summary of MEC cell yield within subject.

Animal ID#	Genotype	Sex	Age Group	# recording sessions	# total cells	# total cells MEC	# grid cells	# HD cells	# Non-grid spatial cells
12015	nTG	F	A	5	21	18	0	7	0
12040	APP	M	A	11	35	35	1	5	1
12375	nTG	F	Y	21	114	114	1	35	14
12378	nTG	F	Y	9	28	28	1	12	0
12644	nTG	F	A	7	32	0	0	0	0
12646	APP	F	A	2	3	3	1	3	0
12655	nTG	F	A	5	15	4	0	2	0
12656	nTG	F	A	8	36	0	0	0	0
12746	nTG	F	A	10	36	36	2	20	3
12748	nTG	F	A	33	97	97	16	25	7
12756	nTG	F	A	10	36	8	0	3	4
12757	nTG	F	A	18	64	55	0	20	4
12758	APP	F	A	24	112	40	1	17	0
12759	APP	F	A	23	70	70	0	30	3
12784	APP	M	A	18	81	81	1	26	7
12785	APP	M	A	22	32	32	2	11	2
12786	nTG	M	A	18	60	60	1	9	7
12787	nTG	M	A	24	153	91	1	13	9
12788	APP	M	A	7	9	7	0	4	0
12790	APP	M	A	18	79	79	2	27	2
12791	nTG	M	A	38	192	192	1	49	17
12792	APP	M	A	14	31	31	1	11	1
12794	nTG	M	A	14	29	29	0	7	1
13530	APP	F	Y	10	29	5	0	3	0
13532	APP	F	Y	28	117	117	4	38	10
13534	APP	F	Y	30	142	142	27	21	31
13601	nTG	F	Y	18	67	7	0	0	0
13630	nTG	M	Y, A	33	260	260	79	45	29
13631	nTG	M	Y	16	95	95	0	38	6
13683	APP	F	A	12	64	64	1	17	3
13781	nTG	M	A	16	46	16	5	4	2
13782	nTG	M	A	26	168	168	31	54	20
13783	nTG	M	A	23	104	104	7	20	6

13784	nTG	M	A	14	65	11	1	2	1
13791	APP	F	A	14	43	43	0	5	0
13792	APP	F	A	13	40	40	0	5	2
13794	APP	F	Y	17	63	63	0	24	7
13795	APP	M	Y	21	91	64	0	15	3
13798	nTG	M	Y	19	106	106	7	16	10
13799	APP	M	Y	25	86	86	31	13	3
13827	nTG	M	Y	17	84	84	0	36	4
13828	APP	M	A	19	52	52	8	17	0
13884	nTG	F	Y, A	22	57	29	1	12	2
13885	nTG	F	Y, A	25	87	87	6	23	7
13894	APP	F	A	17	86	86	2	12	14
13895	nTG	F	Y	18	63	63	1	17	0
13927	APP	F	A	23	97	97	0	44	2
13928	nTG	F	Y	10	20	20	0	10	0
13931	APP	F	A	16	53	53	1	12	10
14012	APP	F	A	13	40	40	0	18	0
14014	APP	M	A	15	62	62	1	12	1
14015	APP	M	A	15	80	80	0	30	4
14020	APP	F	A	12	47	47	0	20	3
14117	APP	F	A	12	29	29	0	9	2
14118	APP	F	A	12	34	34	6	3	4
14125	APP	M	A	15	59	59	12	10	1
14574	APP	F	Y, A	22	71	71	0	10	11
14593	APP	F	A	20	63	63	6	21	7
14598	APP	M	A	14	57	36	0	20	0
14599	APP	M	A	25	68	68	2	9	8
14623	APP	M	A	25	94	63	1	9	28
14754	APP	M	Y, A	30	175	139	1	31	0
14756	APP	M	Y, A	30	267	267	3	127	26
14757	APP	M	Y, A	34	168	164	8	43	7
14847	nTG	F	Y, A	21	63	63	0	25	1
14849	nTG	M	Y, A	20	95	52	1	16	1
15035	APP	F	Y, A	30	188	188	2	45	22
15036	nTG	F	Y	25	108	64	1	19	10

Supplementary Table 2. Summary of CA1 cell yield within subject.

Animal ID#	Genotype	Sex	# recording sessions	# total cells	# place cells
16129	APP	F	17	58	28
16130	nTG	F	15	74	58
16132	nTG	M	14	60	27
16133	APP	M	14	102	76
16135	nTG	M	24	137	79
16153	nTG	F	2	9	4
16154	APP	F	17	138	45
17624	APP	F	22	53	18
17625	nTG	F	18	87	37
17627	APP	F	19	115	51
17628	nTG	F	18	74	49
17903	APP	M	8	85	33

Supplementary Figure 1

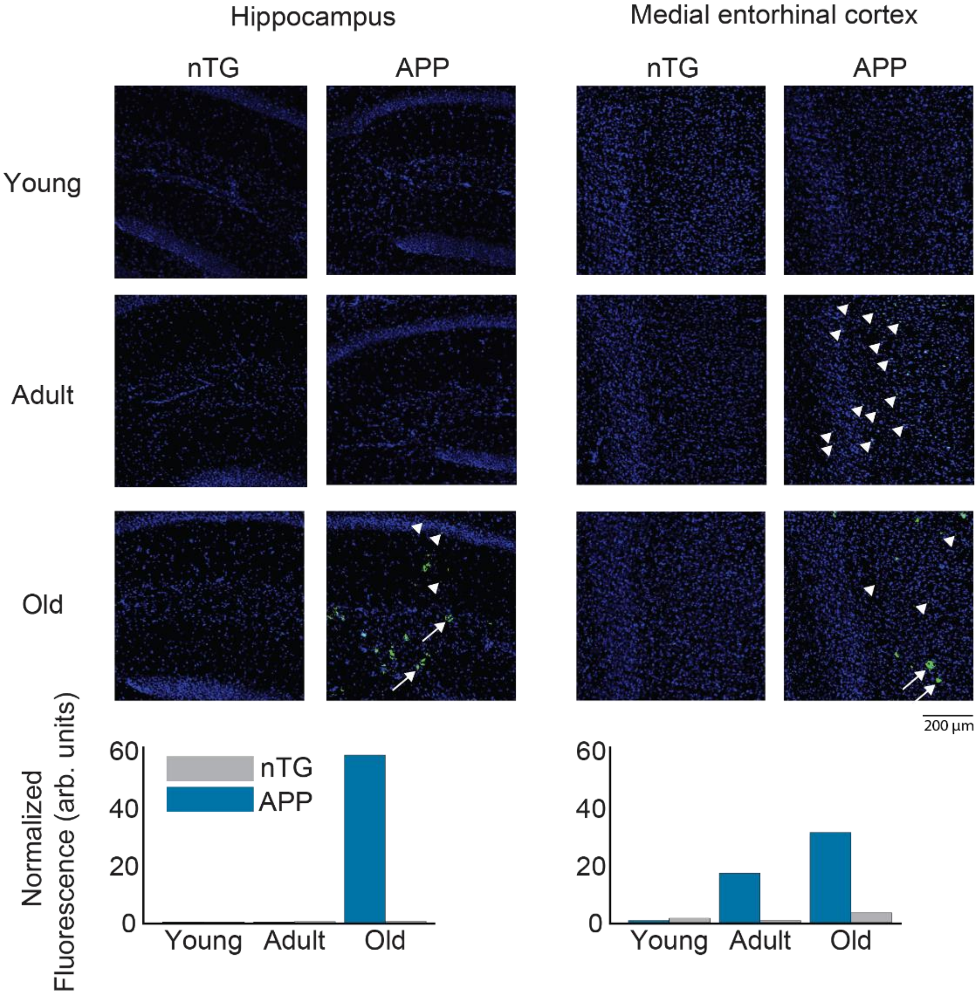

Supplementary Figure 1. Quantification of amyloid-beta plaques in APP mice. Representative examples of magnified brain sections of the hippocampus and medial entorhinal cortex of nTG and APP mice across 3 different age groups: young (3-4.5 mo.), adult (4.5-7 mo.) and old (18 mo.). Arrows and arrowheads indicate the presence of two different kinds of fluorescent morphologies. Adult APP mice have low levels of fluorescence in the medial entorhinal cortex, but the fluorescent signal is intracellular and does not resemble the bigger and widespread morphology observed in the hippocampus and medial entorhinal cortex of old APP mice. The fluorescence signal in adult APP mice might therefore represent early deposition of fibrillar amyloid-beta prior to the formation of mature plaques.

Supplementary Figure 2. Page 1

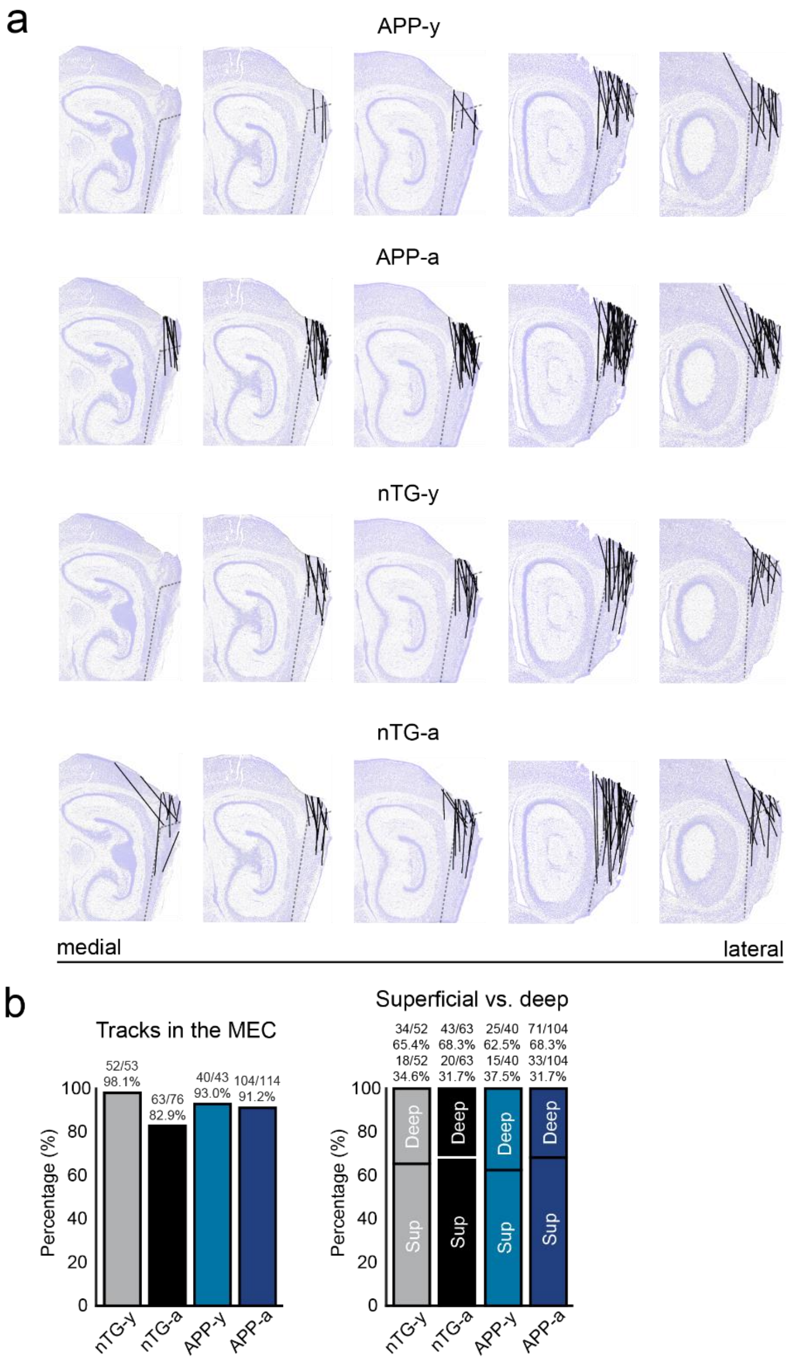

Supplementary Figure 2. Page 2

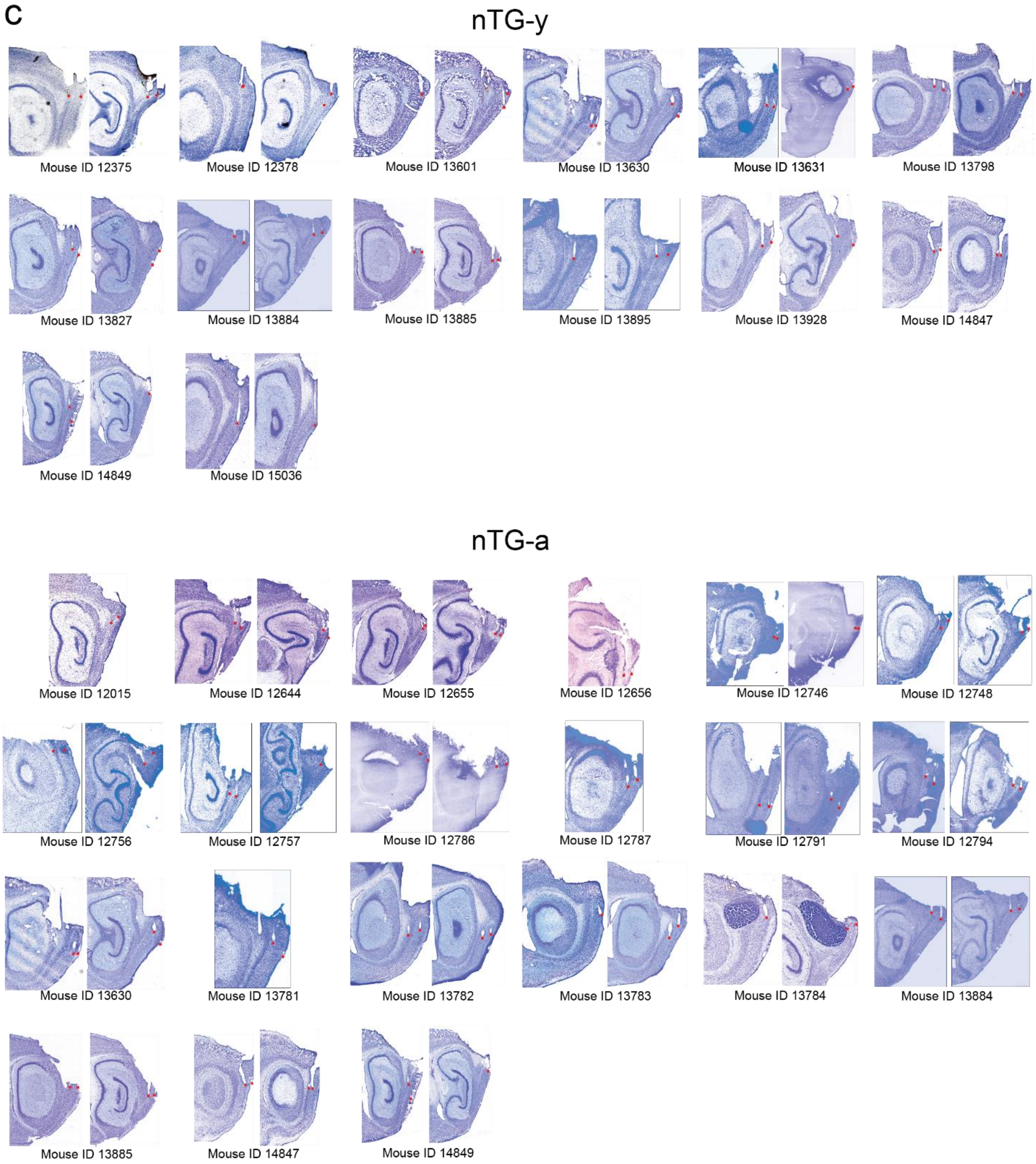

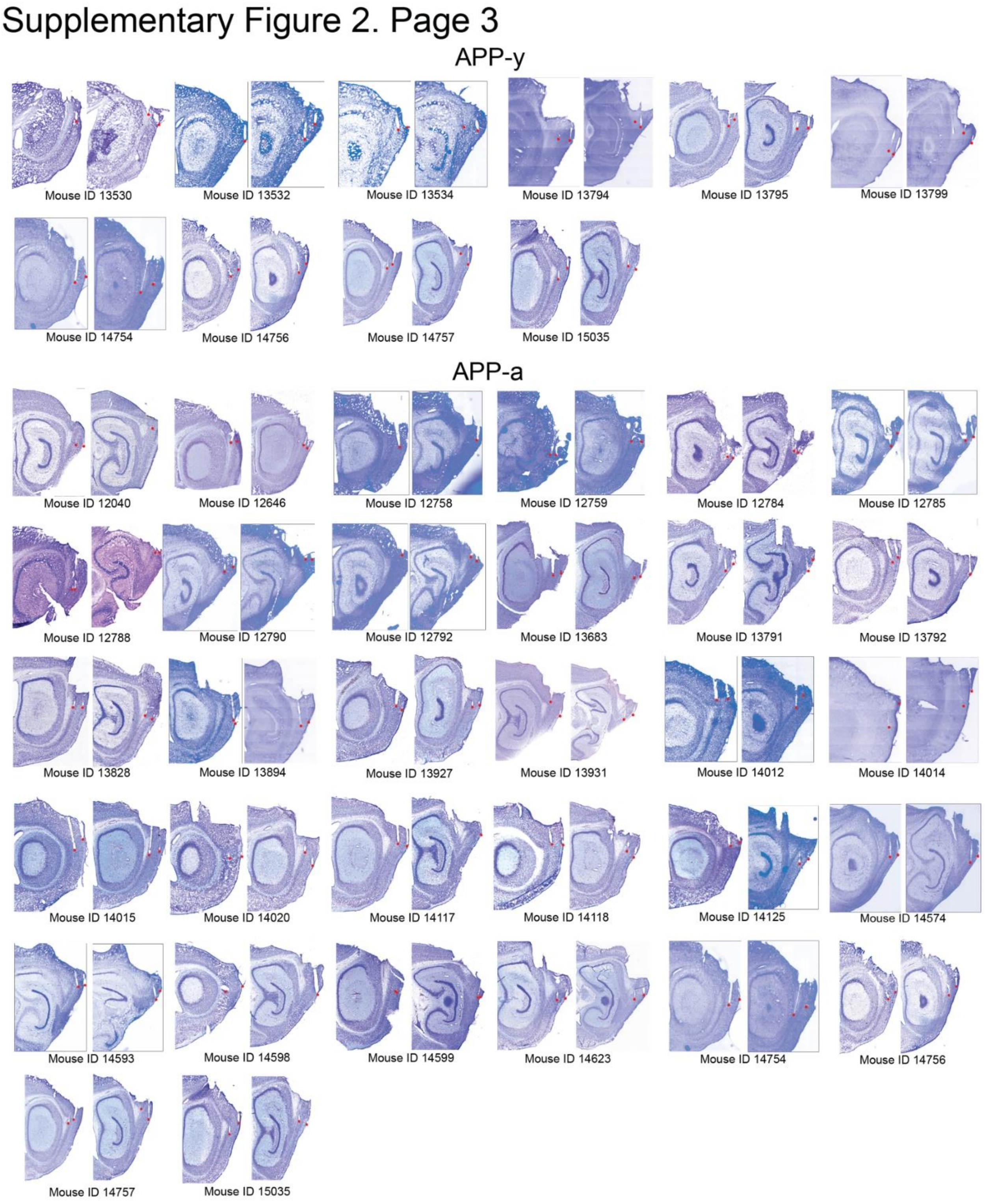

Supplementary Figure 2. Tetrode track histology for MEC recordings. a Averaged sagittal brain sections from the most lateral part of MEC (far right) to the start of parasubiculum (far left).

The individual track locations obtained from each animal were plotted along these sections by experimental group. **b** Quantification of track locations shown in (**a**). (Left) Comparison of the percentage of tetrode tips located in the MEC between groups. (Right) Comparison of the percentage of tetrode tip in either the superficial or deep layers of MEC between groups. **c** Track locations of each animal ordered by genotype and age. The tips of tracks are highlighted with a red dot.

Supplementary Figure 3

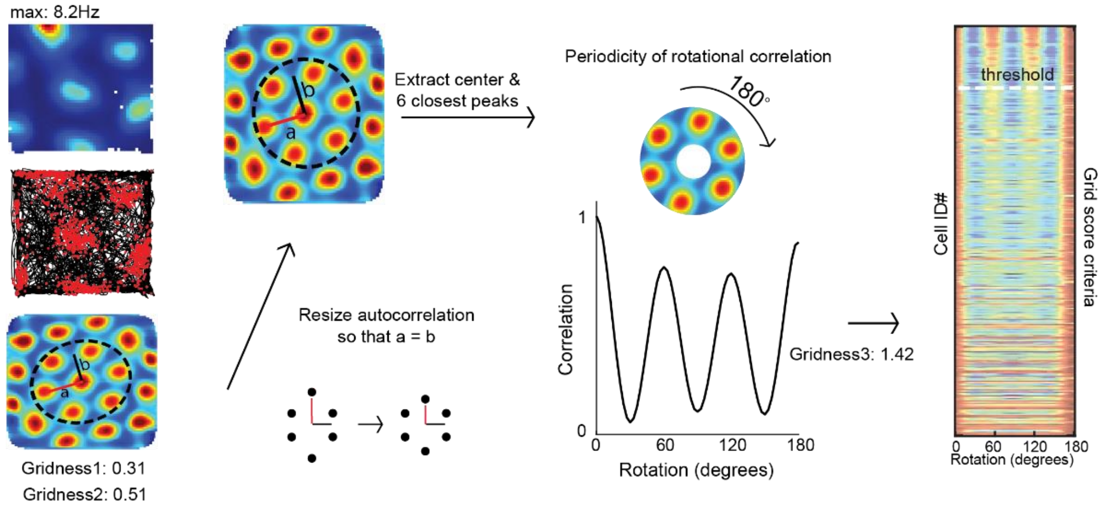

Supplementary Figure 3. Creation of raster plots indicating the strength of rotational correlation of each cell. Autocorrelations of grid cell rate maps were resized to ensure that the major and minor axes, a and b, were equal in length. The resulting image was then rotated 180 degrees to compute a color-coded row indicating the correlation strength at each degree of rotation. These rows were then sorted by decreasing order of grid score in a raster plot.

Supplementary Figure 4

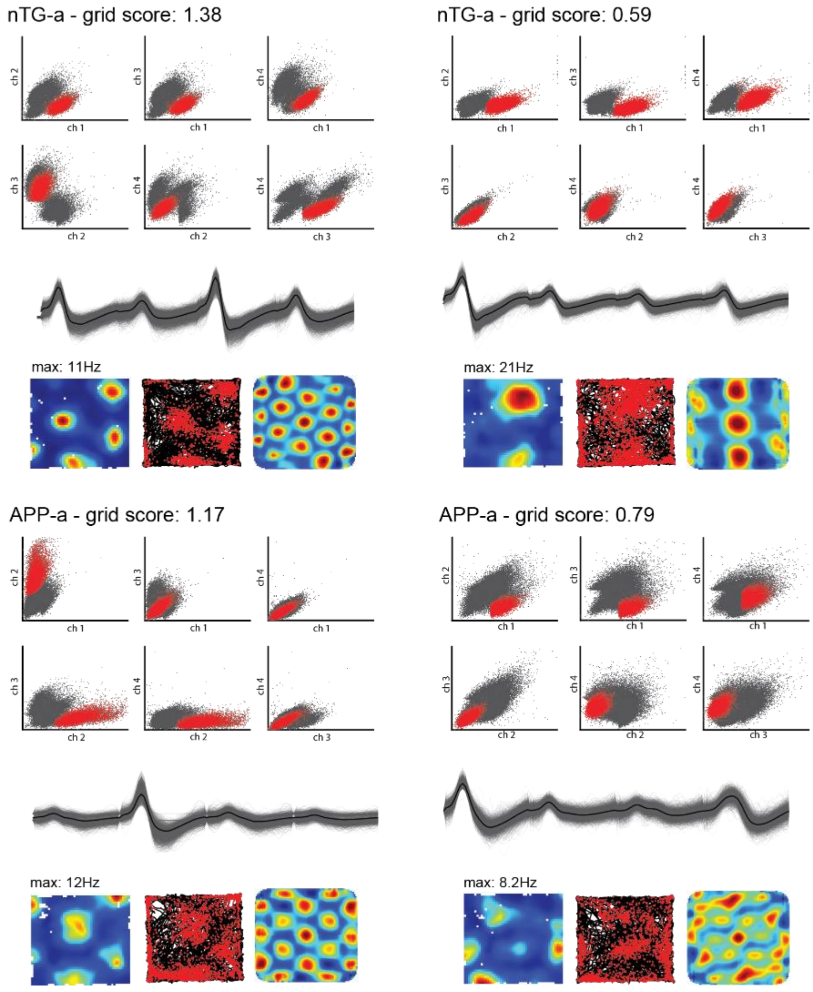

Supplementary Figure 4. Example of well-isolated waveforms of four grid cells recorded in nTG-a and APP-a mice. Cells with a high and low grid score were selected in each group for comparison. Each panel consists of the cell's grid score, the unit location in the six possible conformations of cluster space sorted by waveform amplitude, individual waAveforms recorded across the four recording channels (grey) and the average waveform (black), the cell's rate map, trajectory map and rate map autocorrelation.

Supplementary Figure 5

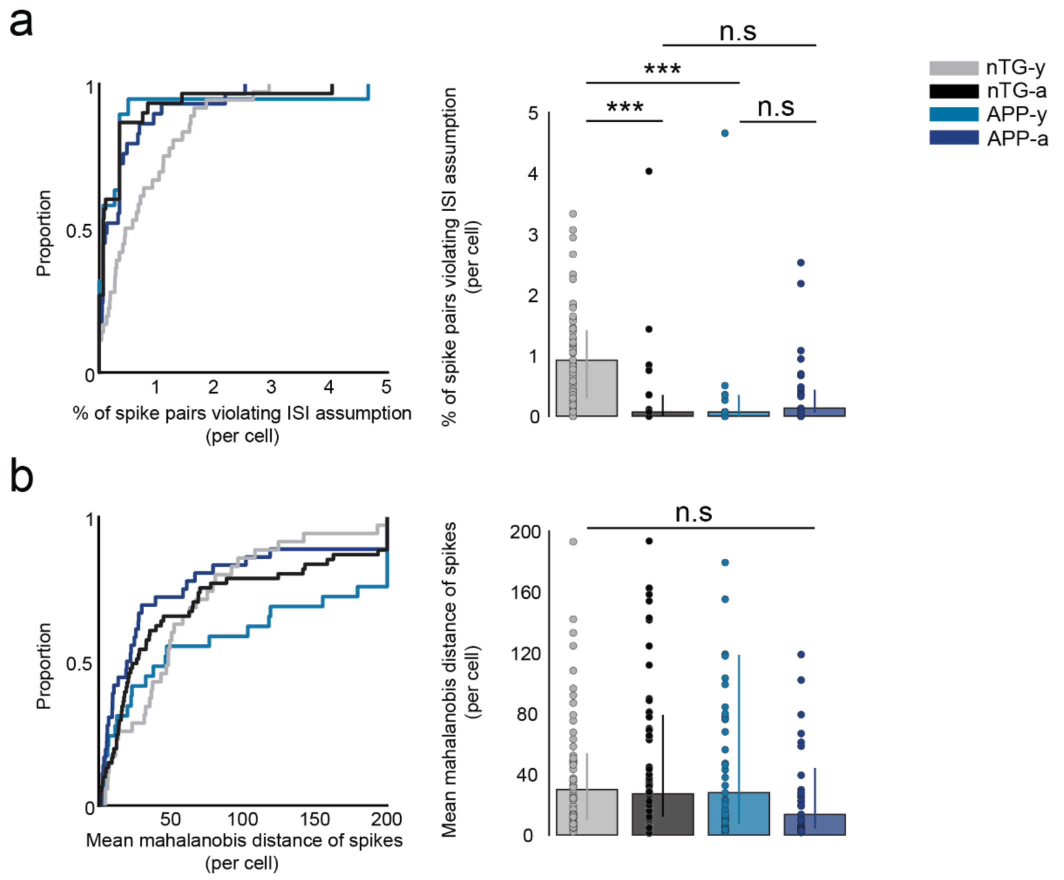

Supplementary Figure 5. Spike isolation quality of grid cells. a Percentage of spike pairs with an interspike interval (ISI) less than 1 millisecond (nTG-y vs. nTG-a: $P = 1.3 \times 10^{-5}$; nTG-y vs. APP-y: $P = 1.2 \times 10^{-4}$; APP-y vs. APP-a: $P = 0.30$; nTG-a vs. APP-a: $P = 0.45$), between groups (cells, $n = 61$ for nTG-y; $n = 30$ for nTG-a; $n = 19$ for APP-y; $n = 30$ for APP-a). These instances are considered non-physiological and may be due to faulty isolation or the presence of noisy spikes. **b** Mean mahalanobis distance of spikes per grid cell (nTG-y vs. nTG-a: $P = 0.65$; nTG-y vs. APP-y: $P = 0.58$; APP-y vs. APP-a: $P = 0.066$; nTG-a vs. APP-a: $P = 0.18$), between groups (cells, $n = 61$ for nTG-y; $n = 97$ for nTG-a; $n = 74$ for APP-y; $n = 45$ for APP-a). nTG-y, non-transgenic young; nTG-a, non-transgenic adult; APP-y, APP young; APP-a, APP adult. Wilcoxon rank sum tests (two-sided) corrected for multiple comparisons using a Bonferroni-Holm correction were applied to analyze the data in Supplementary Fig. 5 **a-b**. Data in bar graphs are presented as median values ± 25th and 75th percentiles; ***$P < 0.001$; n.s, not significant. Source data are provided as a Source Data file.

Supplementary Figure 6

Two-way ANOVA: Grid cells

Source	Sum Square	*df*	MS	*F*	*p*
Age	1.97	1	1.97	28.54	= 1.89e-7
Genotype	0.45	1	0.45	6.54	= 0.011
Interaction	0.89	1	0.89	12.90	= 3.86e-4
Error	19.55	283	0.0069		
Total	22.27	286			

Two-way ANOVA: Head-direction cells

Source	Sum Square	*df*	MS	*F*	*p*
Age	0.19	1	0.19	3.71	= 0.054
Genotype	0.17	1	0.17	3.25	= 0.072
Interaction	0.01	1	0.01	0.2	= 0.65
Error	67.45	1311	0.05		
Total	67.77	1314			

Two-way ANOVA: Non-grid spatial cells

Source	Sum Square	*df*	MS	*F*	*p*
Age	941105	1	941105	0.48	= 0.49
Genotype	933809	1	933809	0.47	= 0.49
Interaction	735051	1	735051	0.37	= 0.54
Error	7.6e8	386	1969180		
Total	7.6e8	389			

Supplementary Figure 6. Spatial tuning of grid cells, but not head-direction cells or non-grid spatial cells, is disrupted across age in APP mice. Two-way unbalanced ANOVAs compare the effects of age, genotype, and interaction on spatial tuning scores for grid cells (grid score), head-direction cells (mean resultant length) and non-grid spatial cells (field size cm^2).

df = degrees of freedom, MS = mean square.

Supplementary Figure 7

a

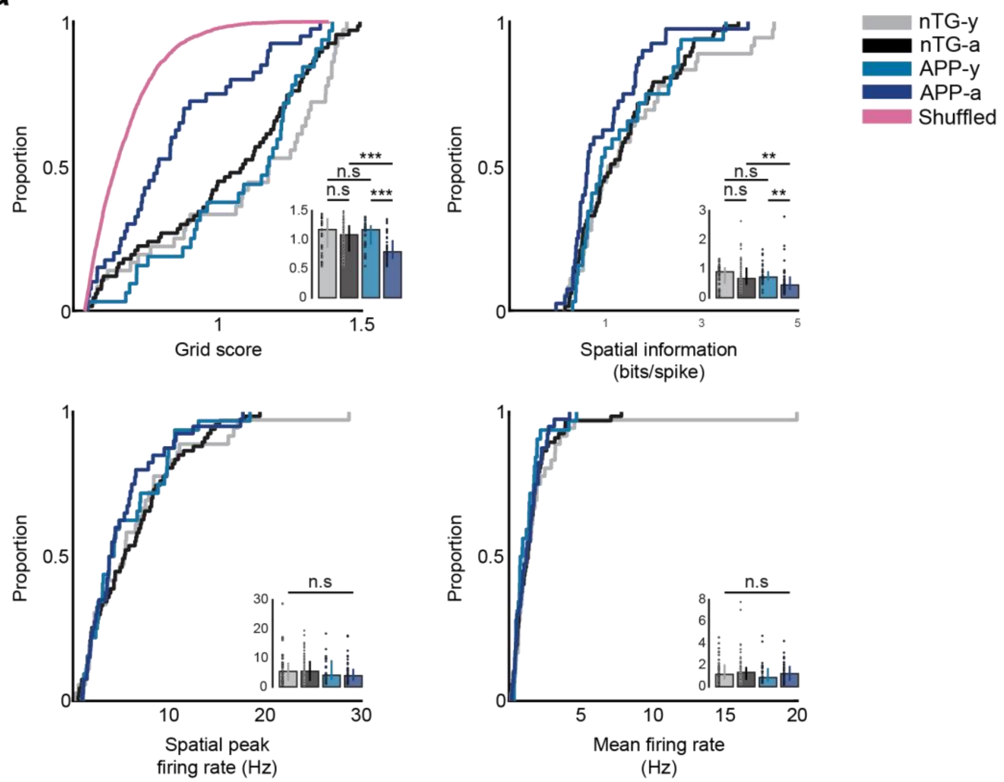

b

Two-way ANOVA: Grid cells

Source	Sum Square	*df*	MS	*F*	*p*
Age	0.90	1	0.90	12.81	= 0.00045
Genotype	0.44	1	0.44	6.31	= 0.013
Interaction	0.28	1	0.28	4.04	= 0.046
Error	12.06	171	0.07		
Total	13.62	174			

Supplementary Figure 7. Grid cell spatial tuning remains impaired in adult APP mice when duplicate cell counts are removed. a Grid score (nTG-y vs. nTG-a: $P = 0.19$; nTG-y vs. APP-y: $P = 0.44$; APP-y vs. APP-a: $P = 4.1 \times 10^{-4}$; nTG-a vs. APP-a: $P = 0.0031$), spatial information (nTG-y vs. nTG-a: $P = 0.73$; nTG-y vs. APP-y: $P = 0.70$; APP-y vs. APP-a: $P = 0.0044$; nTG-a vs. APP-a: $P = 0.0072$), spatial peak firing rate (nTG-y vs. nTG-a: $P = 0.89$; nTG-y vs. APP-y: $P = 0.64$; APP-y vs. APP-a: $P = 0.73$; nTG-a vs. APP-a: $P = 0.22$), and mean firing rate (nTG-y vs. nTG-a: $P = 0.67$; nTG-y vs. APP-y: $P = 0.23$; APP-y vs. APP-a: $P = 0.45$; nTG-a vs. APP-a: $P = 0.80$) between groups (cells, $n = 36$ for nTG-y; $n = 67$ for nTG-a; $n = 32$ for APP-y; $n = 40$ for APP-a). **b** Two-way unbalanced ANOVAs comparing the effects of age, genotype, and interaction on grid scores for grid cells. df= degrees of freedom, MS = mean square. nTG-y, non-transgenic

young; nTG-a, non-transgenic adult; APP-y, APP young; APP-a, APP adult. Wilcoxon rank sum tests (two-sided) corrected for multiple comparisons using a Bonferroni-Holm correction were applied to analyze the data in Supplementary Fig. 7 **a**. Data in bar graphs are presented as median values ± 25th and 75th percentiles; $**P < 0.01$, $***P < 0.001$; n.s, not significant. Source data are provided as a Source Data file.

Supplementary Figure 8

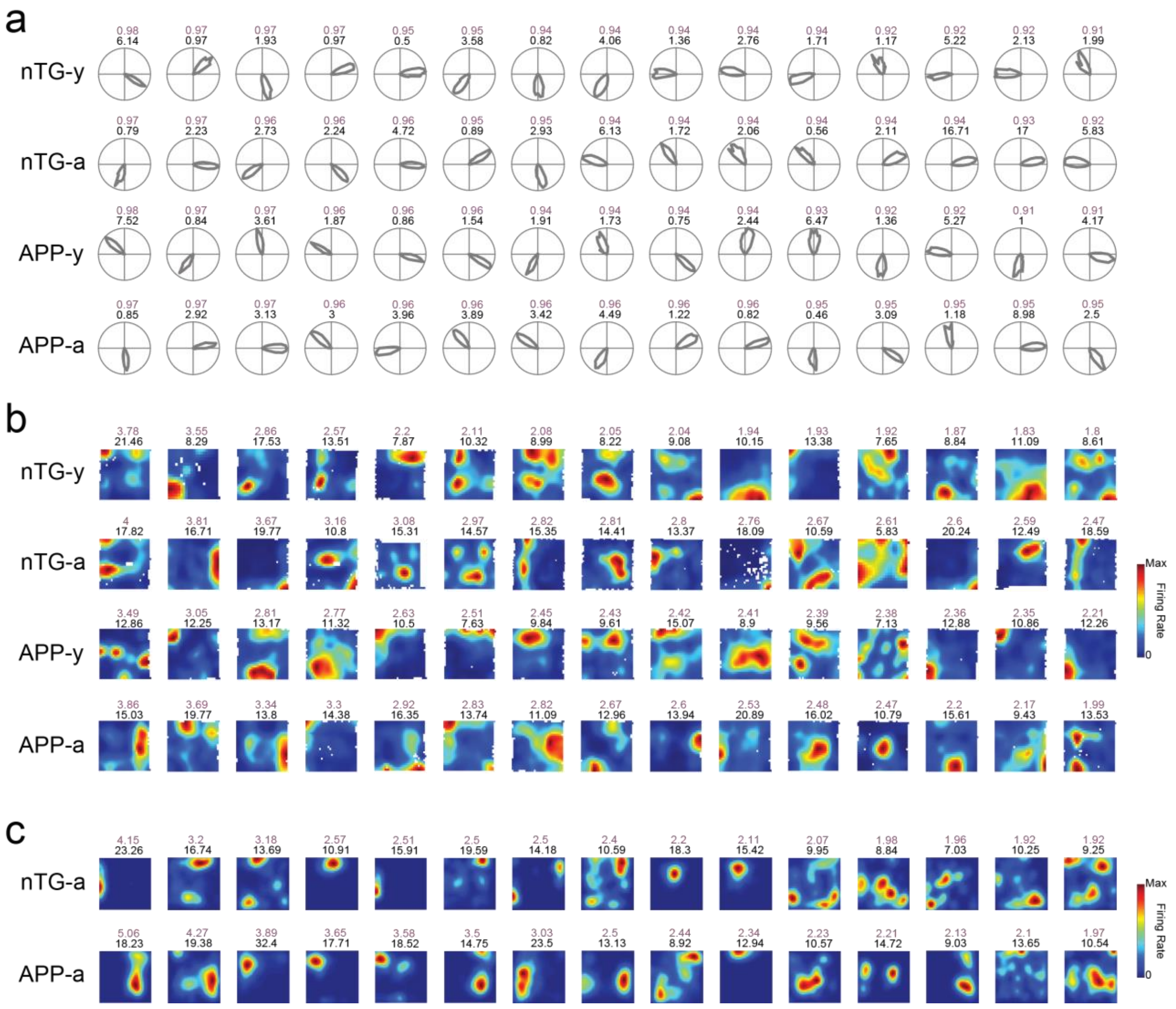

Supplementary Figure 8. Top 15 quality cells across groups. a Polar rate maps for head direction cells from each experimental group. Each row includes 15 head direction cells with the highest mean resultant scores sorted in descending order. The mean resultant length (light purple) and peak firing rate (Hz) (black) of each cell are indicated on top of their respective rate maps. **b** Same as (**a**) but for non-grid spatially-tuned cells. Cells are sorted by the highest spatial information scores in descending order. The spatial information (light purple) and peak firing rate (Hz) (black) of each cell are indicated on top of their respective rate maps. **c** Same as (**a**) but for place cells. Cells are sorted by the highest spatial information scores in descending order. The spatial information (light purple) and peak firing rate (Hz) (black) of each cell are indicated on top of their respective rate maps.

Supplementary Figure 9

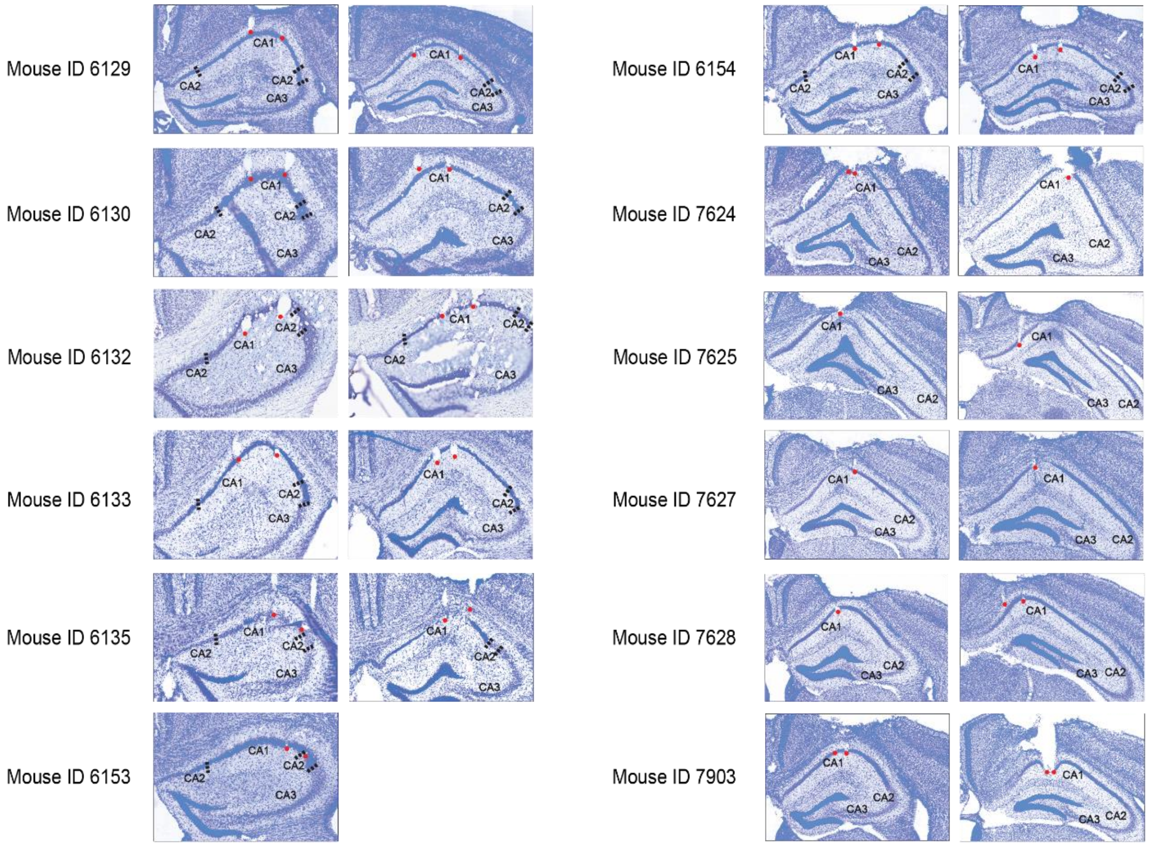

Supplementary Figure 9. Tetrode track histology for CA1 recordings. Track tips in each animal are shown in red dots.

Supplementary Figure 10

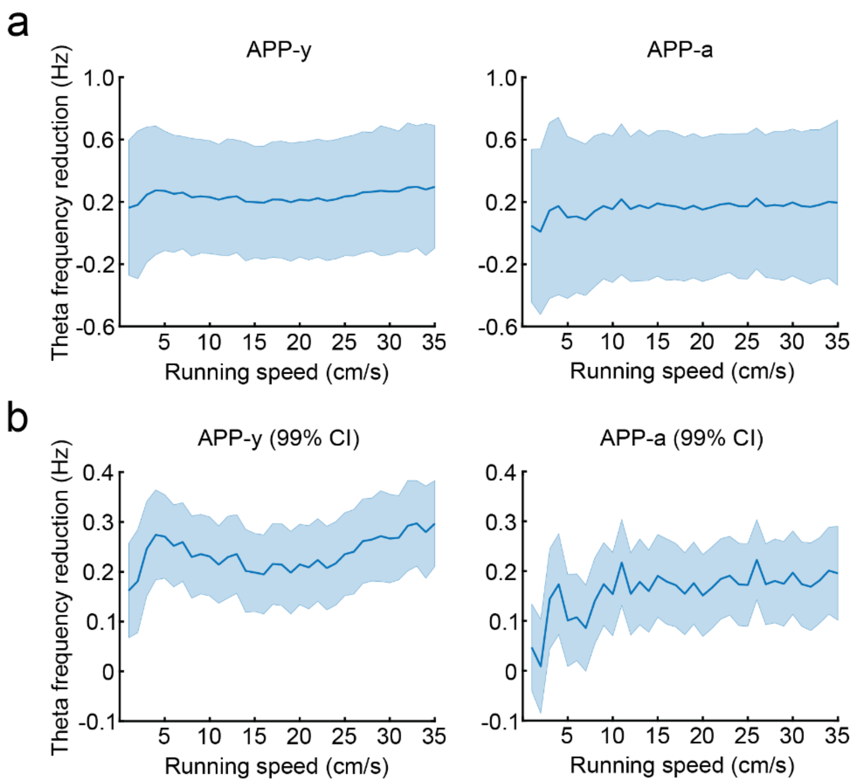

Supplementary Figure 10. The magnitude of theta frequency reduction in APP mice is roughly equal across all running speeds. a Reduction of theta frequency in APP-y (left) and APP-a (right) mice across running speeds (APP mice theta frequencies were subtracted from age-matched non-transgenic counterparts). Data are presented as mean values ± standard deviation. **b** Same as (**a**) but data are presented as mean values ± 99% confidence intervals.

Supplementary Figure 11

a

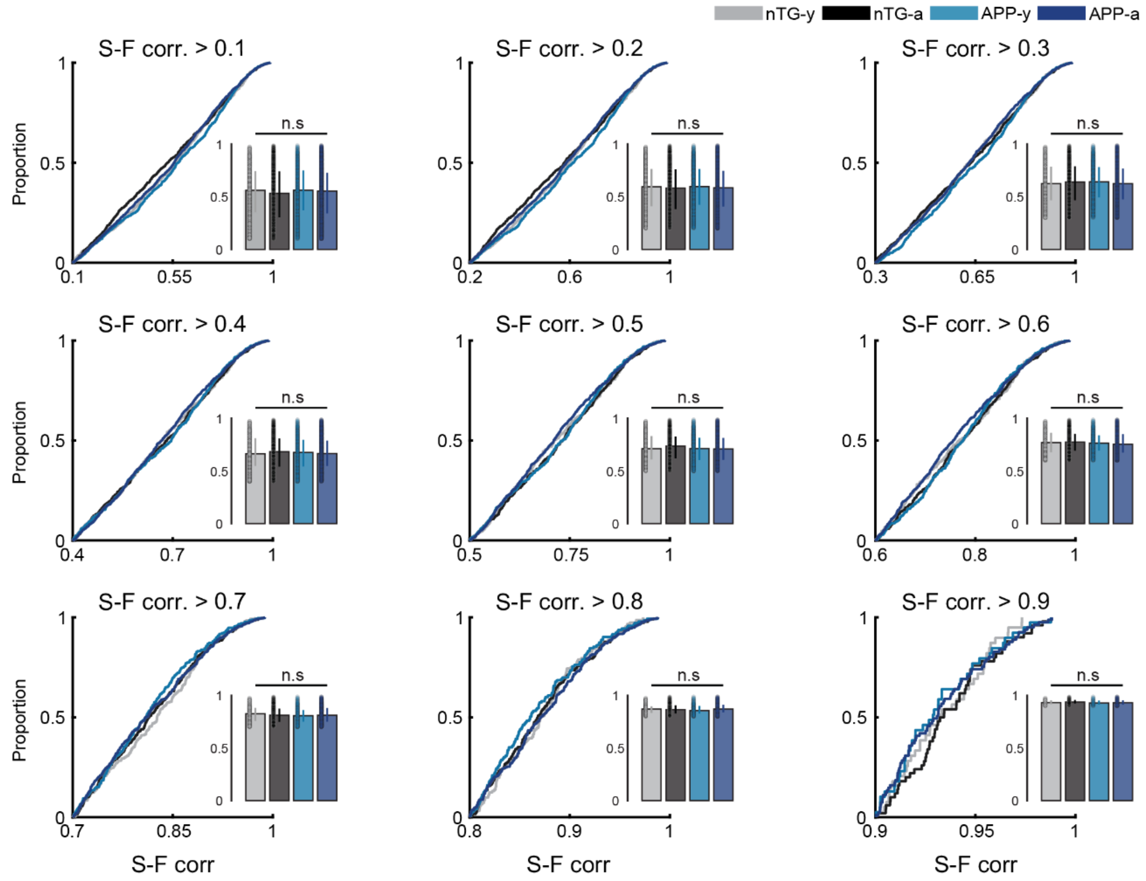

b

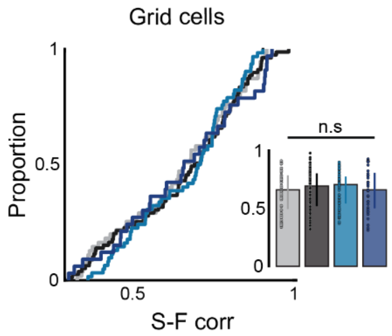

c

Two-way ANOVA: S-F corr. for grid cells

Source	Sum Square	*df*	MS	*F*	*p*
Age	0.00065	1	0.00065	0.02	= 0.88
Genotype	0.0034	1	0.0034	0.11	= 0.74
Interaction	0.00512	1	0.00512	0.17	= 0.68
Error	6.71	225	0.0298		
Total	6.72	228			

Supplementary Figure 11. Intact speed coding in APP mice. a Running speed vs. firing rate correlation (S-F corr.) of putative speed cells. Cells were selected via a S-F corr. threshold ranging from 0.1-0.9.

0.1: nTG-y-nTG-a: $P = 0.17$; nTG-y-APP-y: $P = 0.69$; APP-y-APP-a: $P = 0.23$; nTG-a-APP-a: $P = 0.42$; $n = 772$ nTG-y; 949 nTG-a; 686 APP-y; 1515 APP-a

0.2: nTG-y-nTG-a: P = 0.21; nTG-y-APP-y: P = 0.72; APP-y-APP-a: P = 0.16; nTG-a-APP-a: P = 0.70; n = 696 nTG-y; 846 nTG-a; 619 APP-y; 1368 APP-a

0.3: nTG-y-nTG-a: P = 0.71; nTG-y-APP-y: P = 0.41; APP-y-APP-a: P = 0.23; nTG-a-APP-a: P = 0.47; n = 629 nTG-y; 725 nTG-a; 552 APP-y; 1206 APP-a

0.4: nTG-y-nTG-a: P = 0.61; nTG-y-APP-y: P = 0.65; APP-y-APP-a: P = 0.93; nTG-a-APP-a: P = 0.29; n = 537 nTG-y; 620 nTG-a; 493 APP-y; 1033 APP-a

0.5: nTG-y-nTG-a: P = 0.42; nTG-y-APP-y: P = 0.49; APP-y-APP-a: P = 0.78; nTG-a-APP-a: P = 0.15; n = 442 nTG-y; 507 nTG-a; 407 APP-y; 859 APP-a

0.6: nTG-y-nTG-a: P = 0.64; nTG-y-APP-y: P = 0.74; APP-y-APP-a: P = 0.39; nTG-a-APP-a: P = 0.06; n = 340 nTG-y; 400 nTG-a; 308 APP-y; 658 APP-a

0.7: nTG-y-nTG-a: P = 0.32; nTG-y-APP-y: P = 0.06; APP-y-APP-a: P = 0.40; nTG-a-APP-a: P = 0.86; n = 238 nTG-y; 297 nTG-a; 224 APP-y; 447 APP-a

0.8: nTG-y-nTG-a: P = 0.53; nTG-y-APP-y: P = 0.13; APP-y-APP-a: P = 0.06; nTG-a-APP-a: P = 0.35; n = 142 nTG-y; 167 nTG-a; 119 APP-y; 244 APP-a

0.9: nTG-y-nTG-a: P = 0.54; nTG-y-APP-y: P = 0.72; APP-y-APP-a: P = 0.99; nTG-a-APP-a: P = 0.22; n = 35 nTG-y; 44 nTG-a; 30 APP-y; 80 APP-a

b Grid cell S-F corr. (nTG-y-nTG-a: P = 0.65; nTG-y-APP-y: P = 0.66; APP-y-APP-a: P = 0.99; nTG-a-APP-a: P = 0.98) between groups (n = 55 nTG-y; 79 nTG-a; 62 APP-y; 33 APP-a). **c** Two-way unbalanced ANOVA comparing the effects of age and genotype on S-F corr. of grid cells. df = degrees of freedom, MS = mean square. Wilcoxon rank sum tests (two-sided) with Bonferroni-Holm's correction were applied to Supplementary Fig. 11 **a-b**. Data in bar graphs are presented as medians ± 25th and 75th percentiles; n.s, not significant. Source data are provided as a Source Data file.

Supplementary Figure 12

Two-way ANOVA: Mean 2D displacement

**Source**	**Sum Square**	***df***	**MS**	***F***	***p***
Genotype	33.79	1	33.79	8.43	= 0.0038
Cell type	38.57	2	19.29	4.81	= 0.0084
Interaction	34.83	2	17.41	4.34	= 0.013
Error	3296.64	822	4.01		
Total	3410.74	827			

Pairwise comparisons: Tukey's test

		95% C.I. for true mean diff.		
Comparisons	Mean diff.	LL	UL	*p*
**nTG-a grid - APP-a grid**	**-1.23**	**-2.23**	**-0.23**	**0.0064**
nTG-a grid - nTG-a nongrid	-0.69	-1.56	0.17	0.2
nTG-a grid - APP-a nongrid	-0.79	-1.58	0.00	0.049
nTG-a grid - nTG-a place	-1.08	-1.77	-0.39	0.00012
nTG-a grid - APP-a place	-1.18	-1.87	-0.49	1.60E-05
APP-a grid - nTG-a nongrid	0.54	-0.50	1.57	0.68
APP-a grid - APP-a nongrid	0.44	-0.54	1.41	0.8
APP-a grid - nTG-a place	0.15	-0.75	1.04	1
APP-a grid - APP-a place	0.05	-0.84	0.94	1
**nTG-a nongrid - APP-a nongrid**	**-0.10**	**-0.93**	**0.73**	**1**
nTG-a nongrid - nTG-a place	-0.39	-1.13	0.35	0.66
nTG-a nongrid - APP-a place	-0.49	-1.22	0.25	0.41
APP-a nongrid - nTG-a place	-0.29	-0.94	0.36	0.8
APP-a nongrid - APP-a place	-0.39	-1.03	0.26	0.53
**nTG-a place - APP-a place**	**-0.10**	**-0.61**	**0.42**	**0.99**

Supplementary Figure 12. Mean 2D displacement is higher in grid cells, but not non-grid spatially-tuned cells or place cells, in adult APP mice. Two-way unbalanced ANOVAs compare the effects of genotype, cell type, and interaction on the mean 2D displacement scores for grid cells, non-grid spatially-tuned cells and place cells. Grid cells, non-grid spatially-tuned cells and place cells were included in the "Cell type" factor. Pairwise comparisons with Tukey's test at a corrected alpha value of 0.05 are shown at the bottom. The 3 comparisons of interest (nTG-a grid - APP-a grid; nTG-a nongrid - APP-a nongrid; nTG-a place - APP-a place) are bolded.

df = degrees of freedom, MS = mean square.

Supplementary Figure 13. Page 1

a

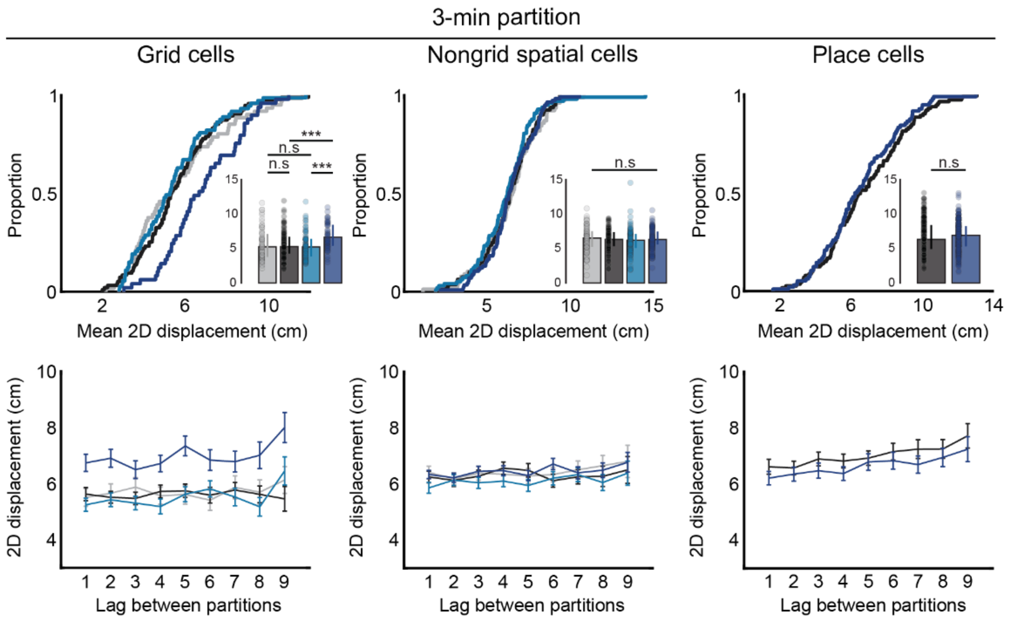

b

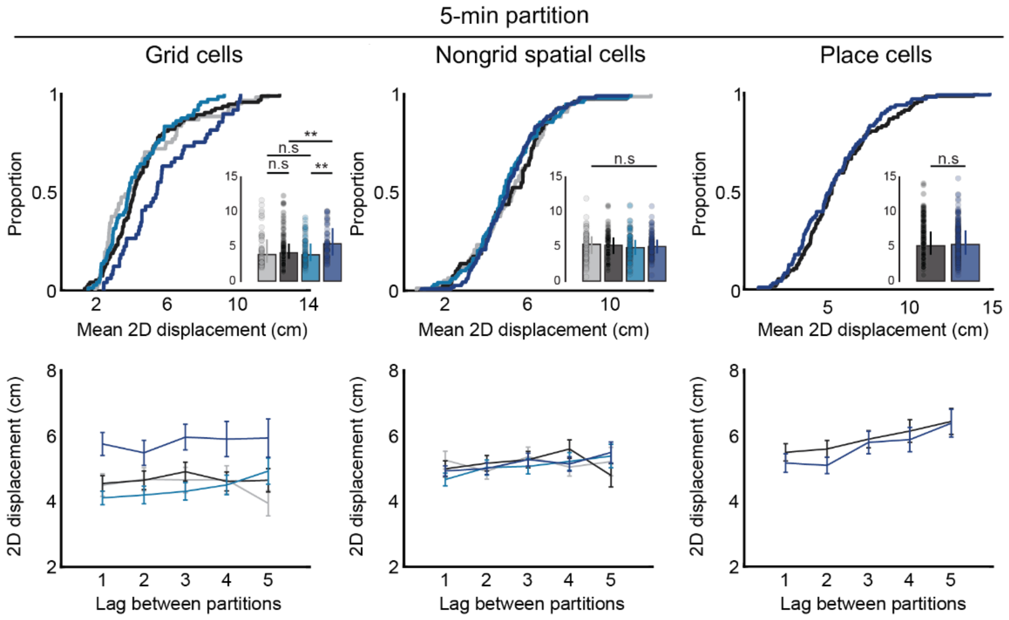

Supplementary Figure 13. Page 2

c

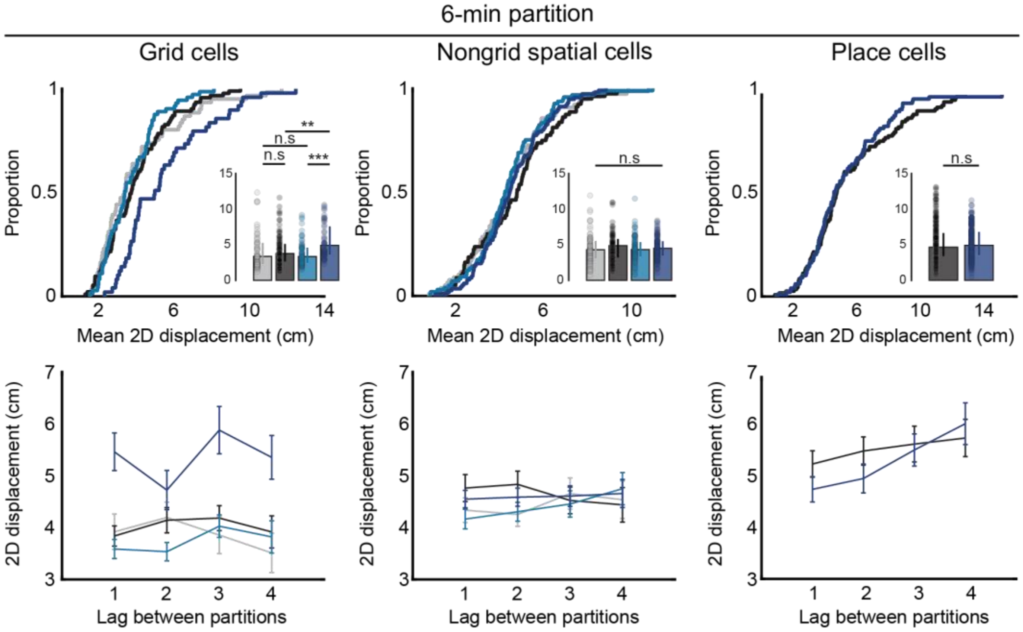

d

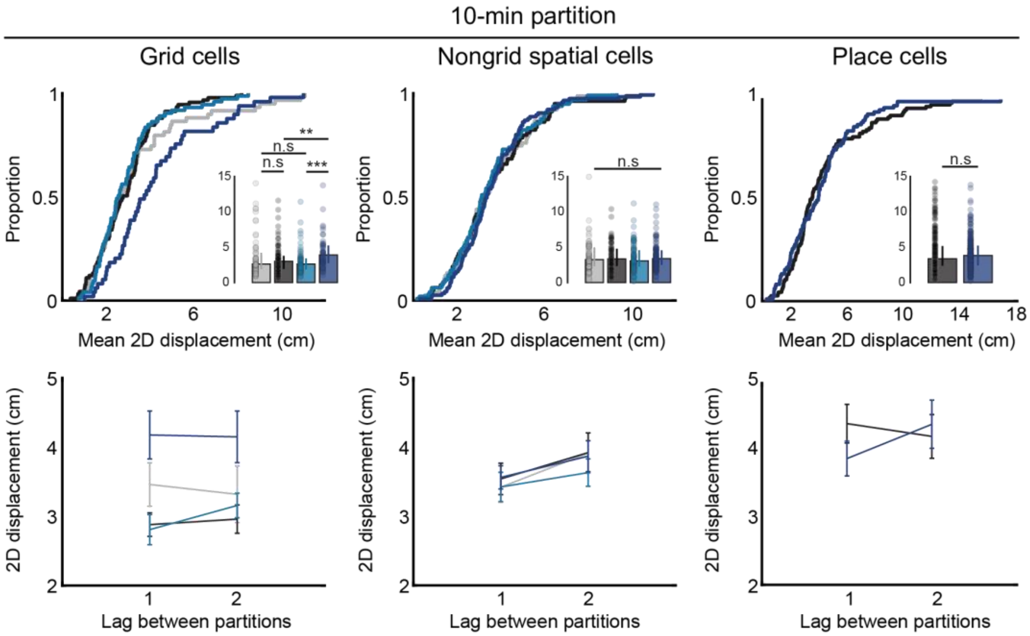

Supplementary Figure 13. Reduced grid cell spatial stability in adult APP mice persists across different partition lengths. **a** 3-minute partition analyses. Two-dimensional displacement of grid cells (nTG-y-nTG-a: P = 0.76; nTG-y-APP-y: P = 0.76; APP-y-APP-a: $P = 9.1 \times 10^{-4}$; nTG-a-APP-a: $P = 3.4 \times 10^{-4}$), non-grid spatially-tuned cells (nTG-y-nTG-a: P = 0.74; nTG-y-

APP-y: $P = 0.24$; APP-y-APP-a: $P = 0.19$; nTG-a-APP-a: $P = 0.87$), and place cells (nTG-a-APP-a: $P = 0.35$) between groups (grid cells, $n = 61$ nTG-y; 95 nTG-a; 73 APP-y; 49 APP-a; non-grid spatially-tuned cells, 77 nTG-y; 80 nTG-a; 98 APP-y; 115 APP-a; place cells, 243 nTG-a; 247 APP-a). (bottom-row) Two-dimensional displacement of grid cells, non-grid spatially-tuned cells and place cells as a function of lags between partitions. Dots indicate mean values and error bars indicate SEM. **b-d** Five-minute (grid cell: nTG-y-nTG-a: $P = 0.35$; nTG-y-APP-y: $P = 0.98$; APP-y-APP-a: $P = 0.0019$; nTG-a-APP-a: $P = 0.01$; non-grid cell: nTG-y-nTG-a: $P = 0.92$; nTG-y-APP-y: $P = 0.35$; APP-y-APP-a: $P = 0.65$; nTG-a-APP-a: $P = 0.58$; place cell: nTG-a-APP-a: $P = 0.67$), 6-minute (grid cell: nTG-y vs. nTG-a: $P = 0.39$; nTG-y vs. APP-y: $P = 1$; APP-y vs. APP-a: $P = 6.0 \times 10^{-5}$; nTG-a vs. APP-a: $P = 0.0025$; non-grid cell: nTG-y vs. nTG-a: $P = 0.31$; nTG-y vs. APP-y: $P = 0.96$; APP-y vs. APP-a: $P = 0.41$; nTG-a vs. APP-a: $P = 0.67$; place cell: nTG-a vs. APP-a: $P = 0.47$) and 10-minute (grid cell: nTG-y vs. nTG-a: $P = 0.93$; nTG-y vs. APP-y: $P = 0.53$; APP-y vs. APP-a: $P = 8.4 \times 10^{-4}$; nTG-a vs. APP-a: $P = 0.0026$; non-grid cell: nTG-y vs. nTG-a: $P = 0.92$; nTG-y vs. APP-y: $P = 0.65$; APP-y vs. APP-a: $P = 0.35$; nTG-a vs. APP-a: $P = 0.79$; place cell: nTG-a vs. APP-a: $P = 0.61$) partitions between groups. Wilcoxon rank sum tests (two-sided) corrected for multiple comparisons using Bonferroni-Holm's correction were applied to Supplementary Fig. 13 **a-d**. Data in bar graphs are presented as median values ± 25th and 75th percentiles; $^{**}P < 0.01$, $^{***}P < 0.001$; n.s, not significant. Source data are provided as a Source Data file.

Supplementary Figure 14

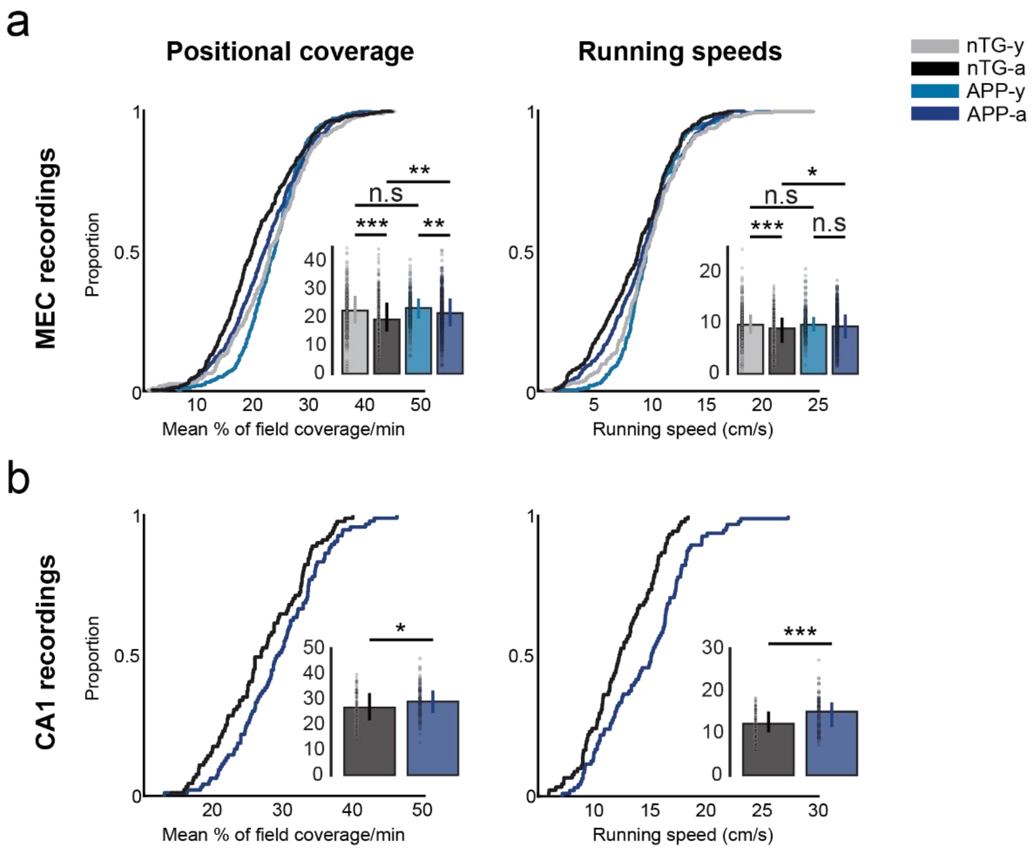

Supplementary Figure 14. Positional coverage and running speeds across groups for MEC and CA1 recordings. a Mean % of the environment covered per minute (nTG-y vs. nTG-a: $P = 3.3 \times 10^{-5}$; nTG-y vs. APP-y: $P = 0.51$; APP-y vs. APP-a: $P = 0.0053$; nTG-a vs. APP-a: $P = 0.0041$), and average running speed (nTG-y vs. nTG-a: $P = 9.1 \times 10^{-4}$; nTG-y vs. APP-y: $P = 0.91$; APP-y vs. APP-a: $P = 0.099$; nTG-a vs. APP-a: $P = 0.047$) between groups (recording session, $n = 270$ for nTG-y; $n = 302$ for nTG-a; $n = 253$ for APP-y; $n = 492$ for APP-a) for MEC recordings. **b** Mean % of the environment covered per minute (nTG-a vs. APP-a: $P = 0.017$), and average running speed (nTG-a vs. APP-a: $P = 1.4 \times 10^{-4}$) between groups (recording session, $n = 91$ for nTG-a; $n = 96$ for APP-a) for CA1 recordings. nTG-y, non-transgenic young; nTG-a, non-transgenic adult; APP-y, APP young; APP-a, APP adult. Wilcoxon rank sum tests (two-sided) corrected for multiple comparisons using a Bonferroni-Holm correction were applied to analyze the data in Supplementary Fig. 14 **a-b**. Data in bar graphs are presented as median values ± 25th and 75th percentiles; $*P < 0.05$, $**P < 0.01$, $***P < 0.001$; n.s, not significant. Source data are provided as a Source Data file.

Supplementary Figure 15

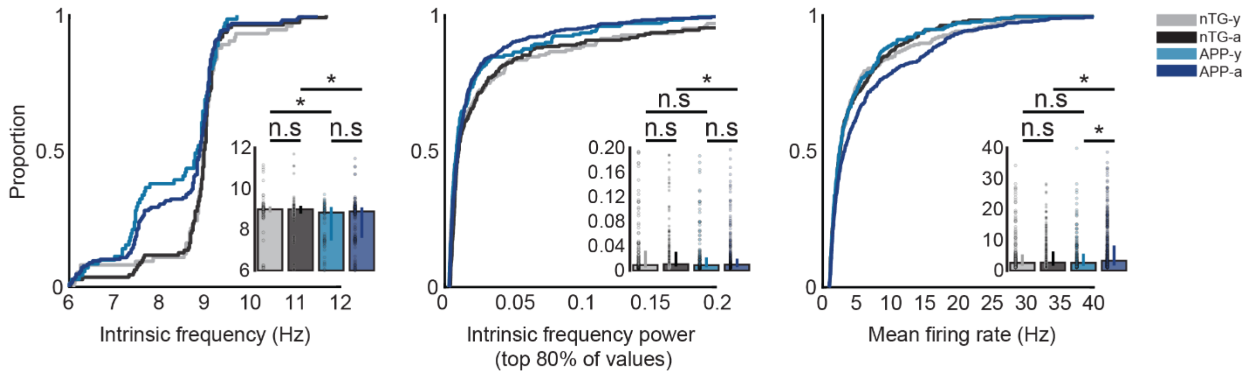

Supplementary Figure 15. Interneuron firing properties. Intrinsic frequency (nTG-y vs. nTG-a: *P* = 0.99; nTG-y vs. APP-y: *P* = 0.038; APP-y vs. APP-a: *P* = 0.49; nTG-a vs. APP-a: *P* = 0.023), intrinsic frequency power (for the top 80% of values) (nTG-y vs. nTG-a: *P* = 0.056; nTG-y vs. APP-y: *P* = 0.58; APP-y vs. APP-a: *P* = 0.38; nTG-a vs. APP-a: *P* = 0.034), and mean firing rate (nTG-y vs. nTG-a: *P* = 0.95; nTG-y vs. APP-y: *P* = 0.82; APP-y vs. APP-a: *P* = 0.034; nTG-a vs. APP-a: *P* = 0.028) of interneurons between groups (cells, intrinsic frequency: *n* = 74 for nTG-y; *n* = 111 for nTG-a; *n* = 71 for APP-y; *n* = 167 for APP-a; intrinsic frequency power: *n* = 164 for nTG-y; *n* = 218 for nTG-a; *n* = 170 for APP-y; *n* = 350 for APP-a; mean firing rate: *n* = 205 for nTG-y; *n* = 272 for nTG-a; *n* = 213 for APP-y; *n* = 437 for APP-a). nTG-y, non-transgenic young; nTG-a, non-transgenic adult; APP-y, APP young; APP-a, APP adult. Wilcoxon rank sum tests (two-sided) corrected for multiple comparisons using a Bonferroni-Holm correction were applied to analyze the data in Supplementary Fig. 15. Data in bar graphs are presented as median values ± 25th and 75th percentiles; *$P < 0.05$; n.s, not significant. Source data are provided as a Source Data file.

Supplementary Figure 16

Two-way ANOVA: Intrinsic frequency (Hz)

Source	Sum Square	*df*	MS	*F*	*p*
Age	0.58	1	0.58	0.60	= 0.44
Genotype	20.42	1	20.42	21.23	= 0.0000054
Interaction	0.61	1	0.61	0.63	= 0.43
Error	403.04	419	0.96		
Total	423.90	422			

Two-way ANOVA: Intrinsic frequency power (top 80% of values)

Source	Sum Square	*df*	MS	*F*	*p*
Age	9 x 10^-5	1	9 x 10^-5	0.03	= 0.85
Genotype	3.74 x 10^-2	1	3.74 x 10^-2	14.72	= 0.0001
Interaction	5.80 x 10^-4	1	5.80 x 10^-4	0.23	= 0.63
Error	2.28	897	2.54 x 10^-3		
Total	2.33	900			

Two-way ANOVA: Mean firing rate (Hz)

Source	Sum Square	*df*	MS	*F*	*p*
Age	147.70	1	147.70	3.06	= 0.081
Genotype	63.60	1	63.60	1.32	= 0.25
Interaction	326.10	1	326.10	6.75	= 0.0095
Error	5.42 x 10^4	1123	48.34		
Total	5.50 x 10^4	1126			

Supplementary Figure 16. Slower intrinsic theta rhythmicity and reduced theta power in APP mice. Two-way unbalanced ANOVAs compare the effects of age, genotype, and interaction on the intrinsic frequency, intrinsic frequency power (for the top 80% of values), and mean firing rates of interneurons between groups.

df = degrees of freedom, MS = mean square.

Supplementary Figure 17

Two-way ANOVA: Grid cell-interneuron synchrony

Source	Sum Square	*df*	MS	*F*	*p*
Age	2.38	1	2.38	0.27	= 0.60
Genotype	173.58	1	173.58	19.8	= 0.000013
Interaction	25.16	1	25.17	2.87	= 0.09
Error	2270.69	259	8.77		
Total	2520.39	262			

Two-way ANOVA: Grid cell-head direction cell synchrony

Source	Sum Square	*df*	MS	*F*	*p*
Age	37.53	1	37.53	7.07	= 0.0083
Genotype	34.11	1	34.11	6.43	= 0.0119
Interaction	16.08	1	16.08	3.03	= 0.08
Error	1289.16	243	5.31		
Total	1356.97	246			

Supplementary Figure 17. Grid cell synchrony with interneurons and head-direction cells are both impaired in APP mice. Two-way unbalanced ANOVAs compare the effects of age, genotype, and interaction on the mean co-activity within a 25 ms time window for grid cell-interneuron pairs, and grid cell-head direction cell pairs.

df = degrees of freedom, MS = mean square.

Supplementary Figure 18

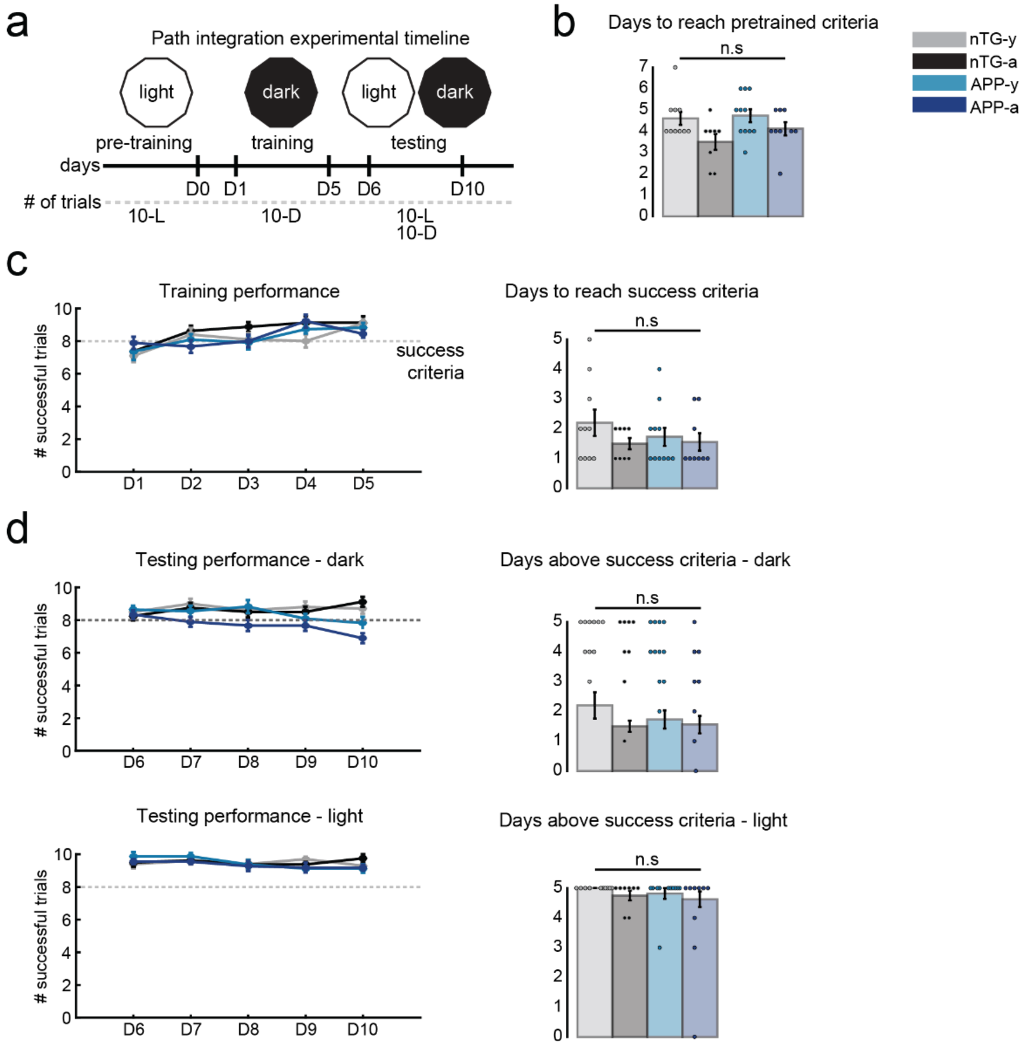

Supplementary Figure 18. Experimental timeline for the path integration task and mouse performance across days. a Experimental timeline for the food-foraging task. Days are indicated as D0, D1, etc. The number of trials that each mouse performed are indicated as 10-L (i.e., 10 light trials) and 10-D (i.e., 10 dark trials). **b** The number of days that it took mice in each group to achieve the success criteria in the pre-training phase (8/10 successful trials) (nTG-y-nTG-a: P = 0.079; nTG-y-APP-y: P = 0.65; APP-y-APP-a: P = 0.24; nTG-a-APP-a: P = 0.28) between groups (mice, n = 10 for nTG-y; n = 8 for nTG-a; n = 11 for APP-y; n = 9 for APP-a). **c** (Left) The number of successful trials achieved by mice across 5 days of training. (Right) The number of days within this training period that mice took to reach the success criteria (nTG-y-nTG-a: P = 0.42; nTG-y-APP-y: P = 0.45; APP-y-APP-a: P = 0.70; nTG-a-APP-a: P = 0.84) across groups (same sample

sizes as in **a**). **d** (Top-Left) Same as (**c**) but across 5 days of testing in dark conditions. (Top-Right) The number of days within this testing period that mice performed at or above the success criteria (nTG-y-nTG-a: P = 0.60; nTG-y-APP-y: P = 0.24; APP-y-APP-a: P = 0.11; nTG-a-APP-a: P = 0.11) across groups (same sample sizes as in **a**). (Bottom-Left) Same as (**c**) but across 5 days of testing in light conditions. (Bottom-Right) The number of days within this testing period that mice performed at or above the success criteria (nTG-y-nTG-a: P = 0.37; nTG-y-APP-y: P = 0.39; APP-y-APP-a: P = 0.21; nTG-a-APP-a: P = 0.59) between groups (same sample sizes as in **a**). nTG-y, non-transgenic young; nTG-a, non-transgenic adult; APP-y, APP young; APP-a, APP adult. Wilcoxon rank sum tests (two-sided) corrected for multiple comparisons using a Bonferroni-Holm correction were applied to analyze the data in Supplementary Fig. 18 **b-d**. Data in line graphs are presented as mean values ± SEM. Data in bar graphs are presented as mean values ± SEM; n.s, not significant. Source data are provided as a Source Data file.

Supplementary Figure 19

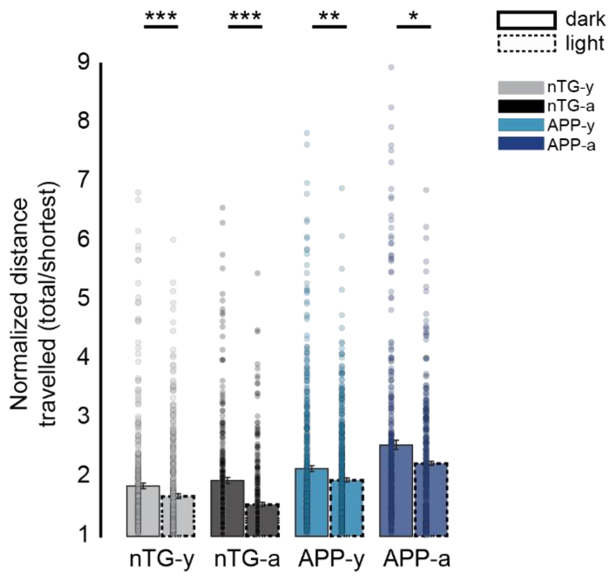

Supplementary Figure 19. Overall navigation ability improves in light trials for all mice. Normalized distance travelled in dark (solid lines) versus light (dashed lines) trials. (nTG-y: $P = 6.9 \times 10^{-9}$; nTG-a: $P = 1.4 \times 10^{-21}$; APP-y: $P = 0.0084$; APP-a: $P = 0.017$) (dark behavior trials, $n = 377$ for nTG-y; $n = 307$ for nTG-a; $n = 500$ for APP-y; $n = 311$ for APP-a; light behavior trials, $n = 426$ for nTG-y; $n = 344$ for nTG-a; $n = 538$ for APP-y; $n = 330$ for APP-a). nTG-y, non-transgenic young; nTG-a, non-transgenic adult; APP-y, APP young; APP-a, APP adult. Wilcoxon rank sum tests (two-sided) were applied to analyze the data in Supplementary Fig. 19. Data are presented as mean values ± SEM; $*P < 0.05$, $**P < 0.01$, $***P < 0.001$; Source data are provided as a Source Data file.

Supplementary Figure 20

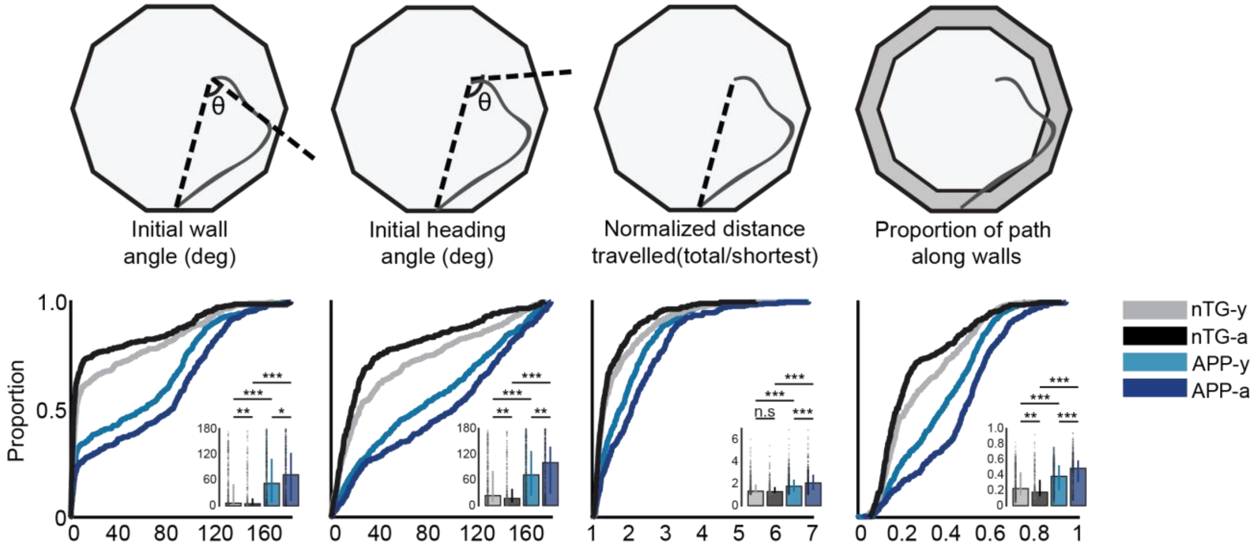

Supplementary Figure 20. APP mice have impaired path integration ability in light conditions. The initial wall angle (nTG-y vs. nTG-a: $P = 0.0075$; nTG-y vs. APP-y: $P = 1.1 \times 10^{-18}$; APP-y vs. APP-a: $P = 0.013$; nTG-a vs. APP-a: $P = 1.5 \times 10^{-32}$), the initial heading angle (nTG-y vs. nTG-a: $P = 0.0029$; nTG-y vs. APP-y: $P = 3.4 \times 10^{-17}$; APP-y vs. APP-a: $P = 0.0044$; nTG-a vs. APP-a: $P = 1.2 \times 10^{-33}$), the normalized distance travelled (nTG-y vs. nTG-a: $P = 0.053$; nTG-y vs. APP-y: $P = 7.7 \times 10^{-12}$; APP-y vs. APP-a: $P = 6.3 \times 10^{-5}$; nTG-a vs. APP-a: $P = 1.2 \times 10^{-26}$), and the proportion of the return path spent along the periphery (nTG-y vs. nTG-a: $P = 0.0015$; nTG-y vs. APP-y: $P = 8.2 \times 10^{-13}$; APP-y vs. APP-a: $P = 9.5 \times 10^{-9}$; nTG-a vs. APP-a: $P = 9.3 \times 10^{-35}$) between groups (behavior trials, $n = 426$ for nTG-y; $n = 344$ for nTG-a; $n = 538$ for APP-y; $n = 330$ for APP-a). nTG-y, non-transgenic young; nTG-a, non-transgenic adult; APP-y, APP young; APP-a, APP adult. Wilcoxon rank sum tests (two-sided) corrected for multiple comparisons using a Bonferroni-Holm correction were applied to analyze the data in Supplementary Fig. 20. Data in bar graphs are presented as median values ± 25th and 75th percentiles; *$P < 0.05$, **$P < 0.01$, ***$P < 0.001$; n.s, not significant. Source data are provided as a Source Data file.

Supplementary Figure 21

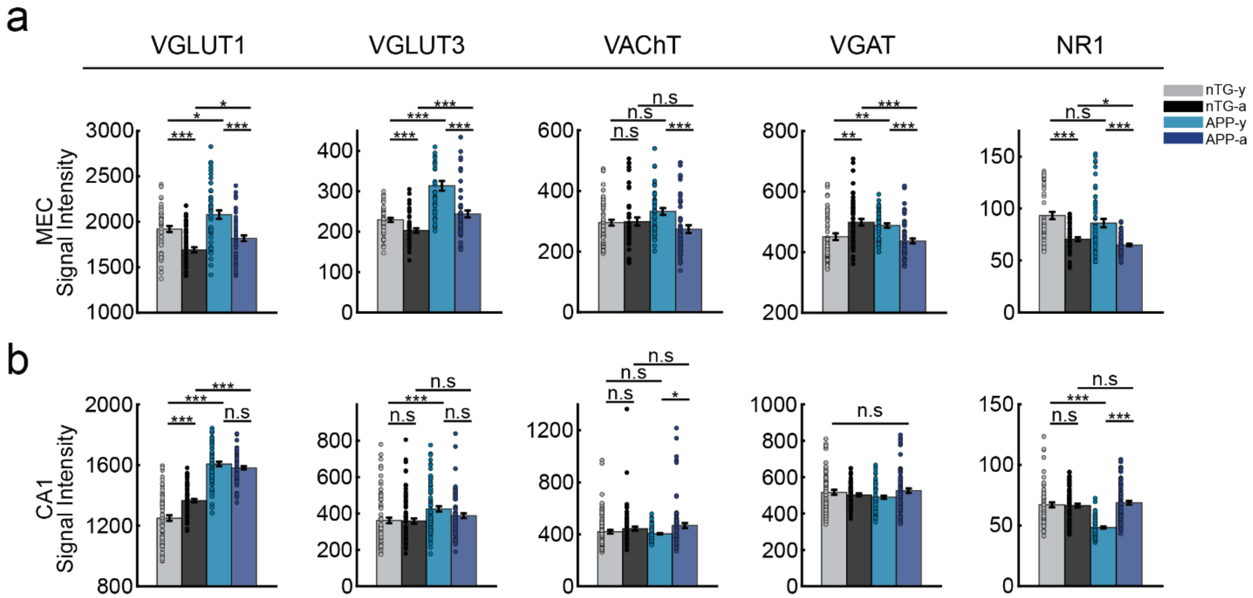

Supplementary Figure 21. Immunoautoradiographic labelling of synaptic markers in MEC and CA1. a Signal intensity of VGLUT1 (nTG-y-nTG-a: $P = 1.3 \times 10^{-5}$; nTG-y-APP-y: $P = 0.014$; APP-y-APP-a: $P = 1.1 \times 10^{-4}$; nTG-a-APP-a: $P = 0.014$; $n = 52$ nTG-y; 53 nTG-a; 48 APP-y; 55 APP-a), VGLUT3 (nTG-y-nTG-a: $P = 1.9 \times 10^{-4}$; nTG-y-APP-y: $P = 1.5 \times 10^{-8}$; APP-y-APP-a: $P = 2.6 \times 10^{-6}$; nTG-a-APP-a: $P = 2.3 \times 10^{-4}$; $n = 52$ nTG-y; 50 nTG-a; 45 APP-y; 51 APP-a), VAChT (nTG-y-nTG-a: $P = 0.87$; nTG-y-APP-y: $P = 0.053$; APP-y-APP-a: $P = 4.5 \times 10^{-4}$; nTG-a-APP-a: $P = 0.051$; $n = 47$ nTG-y; 45 nTG-a; 48 APP-y; 52 APP-a), VGAT (nTG-y-nTG-a: $P = 0.0037$; nTG-y-APP-y: $P = 0.0027$; APP-y-APP-a: $P = 7.4 \times 10^{-6}$; nTG-a-APP-a: $P = 2.1 \times 10^{-5}$; $n = 49$ nTG-y; 50 nTG-a; 46 APP-y; 55 APP-a) and NR1 (nTG-y-nTG-a: $P = 1.6 \times 10^{-5}$; nTG-y-APP-y: $P = 0.063$; APP-y-APP-a: $P = 2.7 \times 10^{-5}$; nTG-a-APP-a: $P = 0.023$; $n = 48$ nTG-y; 53 nTG-a; 51 APP-y; 58 APP-a) in the MEC. **b** Signal intensity of VGLUT1 (nTG-y-nTG-a: $P = 4.6 \times 10^{-6}$; nTG-y-APP-y: $P = 3.9 \times 10^{-21}$; APP-y-APP-a: $P = 0.15$; nTG-a-APP-a: $P = 4.5 \times 10^{-21}$; $n = 79$ nTG-y; 80 nTG-a; 70 APP-y; 79 APP-a), VGLUT3 (nTG-y-nTG-a: $P = 0.76$; nTG-y-APP-y: $P = 5.7 \times 10^{-4}$; APP-y-APP-a: $P = 0.066$; nTG-a-APP-a: $P = 0.10$; $n = 76$ nTG-y; 84 nTG-a; 81 APP-y; 77 APP-a), VAChT (nTG-y-nTG-a: $P = 0.14$; nTG-y-APP-y: $P = 0.30$; APP-y-APP-a: $P = 0.039$; nTG-a-APP-a: $P = 0.39$; $n = 86$ nTG-y; 78 nTG-a; 82 APP-y; 87 APP-a) VGAT (nTG-y-nTG-a: $P = 0.87$; nTG-y-APP-y: $P = 0.33$; APP-y-APP-a: $P = 0.074$; nTG-a-APP-a: $P = 0.35$; $n = 72$ nTG-y; 69 nTG-a; 70 APP-y; 79 APP-a) and NR1 (NR1: nTG-y-nTG-a: $P = 0.71$; nTG-y-APP-y: $P =$

2.1×10^{-14}; APP-y-APP-a: $P = 9.4 \times 10^{-19}$; nTG-a-APP-a: $P = 0.41$; $n = 70$ nTG-y; 82 nTG-a; 72 APP-y; 85 APP-a) in CA1. Wilcoxon rank sum tests (two-sided) corrected for multiple comparisons using Bonferroni-Holm's correction were applied to Supplementary Fig. 21 **a-b**. Data are presented as mean values ± SEM; $^{*}P < 0.05$, $^{**}P < 0.01$, $^{***}P < 0.001$; n.s, not significant. Source data are provided as a Source Data file.

Supplementary Figure 22

a

Linear mixed effects model predicting VGLUT3 expression in the MEC

Model information

Number of observations : 198
Fixed effects coefficients : 3
Random effects coefficients : 48
Covariance parameters : 4

Model fit statistics

AIC : 2071.4
BIC : 2094.4
Log Likelihood : -1028.7
Deviance : 2057.4

Fixed effects coefficients (95% CIs):

Name	Estimate	Std. Error	tStat	DF	*p*	95% CI LL	95% CI UL
Intercept	253.03	18.12	13.96	195	= 3.6e-31	217.29	288.77
Genotype	-60.5	21.78	-2.78	195	= 0.006	-103.45	-17.55
Age	48.23	21.96	2.2	195	= 0.0292	4.93	91.55

Random effects covariance (95% CIs):

Subject(16 levels)	Estimate	95% CI LL	95% CI UL
Intercept	39.48	25.95	60.05
* Genotype	< 0.001	NaN	NaN
* Age	22.15	19.98	24.56

Error	Estimate	95% CI LL	95% CI UL
Residual	39.1	35.29	43.33

b

Linear mixed effects model predicting VGLUT1 expression in CA1

Model information

Number of observations : 308
Fixed effects coefficients : 3
Random effects coefficients : 48
Covariance parameters : 4

Model fit statistics

AIC : 3687.8
BIC : 3713
Log Likelihood : -1836.9
Deviance : 3673.8

Fixed effects coefficients (95% CIs):

Name	Estimate	Std. Error	tStat	DF	*p*	95% CI LL	95% CI UL
Intercept	1590.7	21.47	74.09	305	= 4.8e-197	1548.5	1633
Genotype	-270.68	45.09	-6	305	= 5.5e-9	-359.41	-181.9
Age	-3.91	38.68	-0.1	305	= 0.92	-80.02	72.2

Random effects covariance (95% CIs):

Subject(16 levels)	Estimate	95% CI LL	95% CI UL
Intercept	39.6	17.22	91.03
* Genotype	96.99	30.2	311.5
* Age	61.3	9.05	415.14

Error	Estimate	95% CI LL	95% CI UL
Residual	84.54	80.72	94.93

Supplementary Figure 22. We used the fitlme function in MATLAB to perform linear mixed effects analyses on the relationship between the subjects' genotype (nTG and APP), age

(young and adult) and expression of VGLUT3 and VGLUT1 levels in the MEC and CA1 respectively. The fixed effects of the model comprised genotype and age (without interaction between the two). The random effects of the model comprised random intercepts by-subject, random slopes for the effects of genotype and age by-subject, and independence between the intercepts and slopes. P-values obtained in the model output were considered as the measurements for significance.

a Table shows the model information, statistics of fit, the fixed effects coefficients, and the random effects covariance parameters. In the fixed effects panel, the 'Intercept' refers to the aged APP experimental group; its estimate is the predicted mean VGLUT3 signal intensity. The estimates for genotype and age refer to the predicted slope change from the intercept. Std. Error refers to the standard error associated with the slope. T-values and P-values for the contribution of genotype and age are bolded. **b** Same as (**a**) but for levels of VGLUT1 in CA1.

tStat = T-value, CI = confidence interval, DF = degrees of freedom, LL = lower limit, UL = upper limit.
* = independence between intercepts and slopes.

Supplementary Figure 23

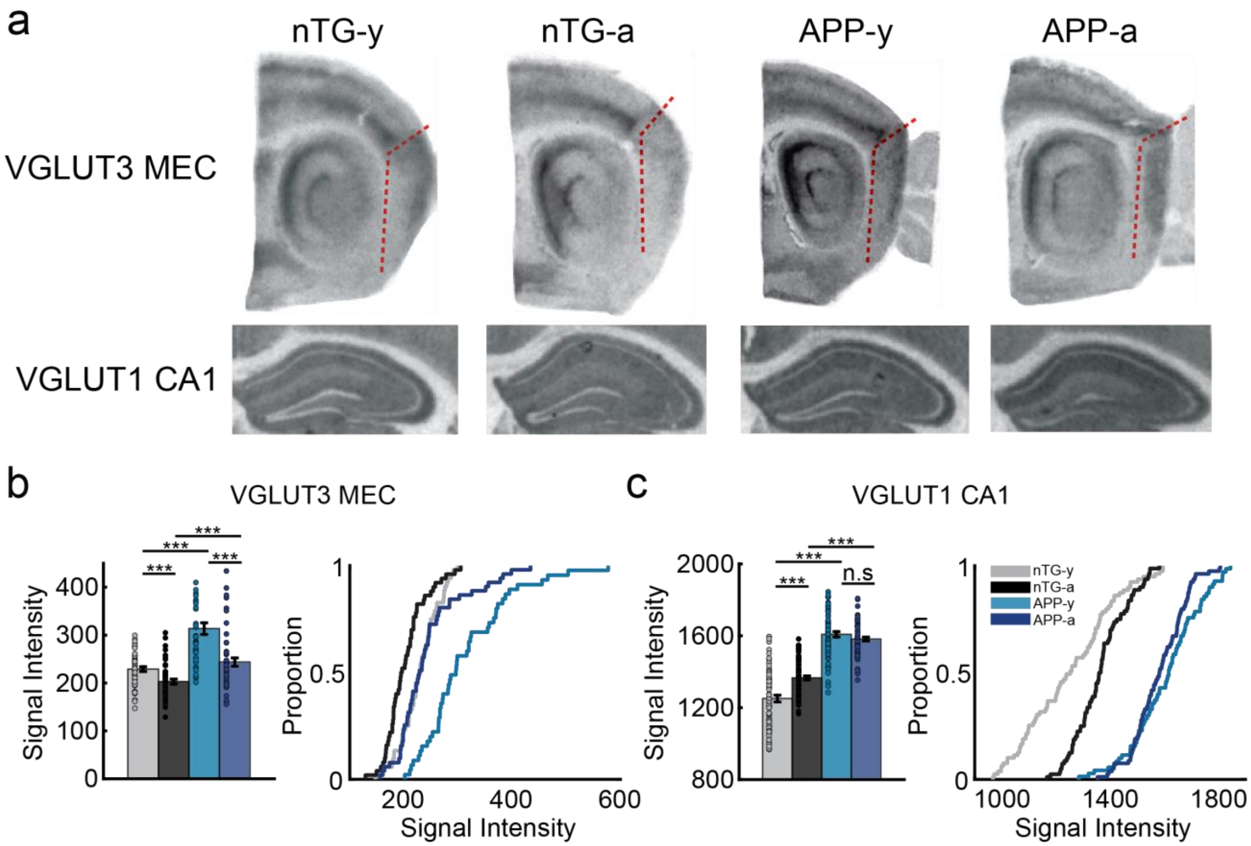

Supplementary Figure 23. Pathological expression of MEC VGLUT3 levels and CA1 VGLUT1 levels in APP mice. a Single slice examples of VGLUT3 and VGLUT1 expression in MEC and CA1 respectively. Darker signals indicate higher marker expression levels. **b** Signal intensity of VGLUT3 levels (nTG-y vs. nTG-a: $P = 1.9 \times 10^{-4}$; nTG-y vs. APP-y: $P = 1.5 \times 10^{-8}$; APP-y vs. APP-a: $P = 2.6 \times 10^{-6}$; nTG-a vs. APP-a: $P = 2.3 \times 10^{-4}$) in the MEC between groups (brain slice, $n = 52$ for nTG-y; $n = 50$ for nTG-a; $n = 45$ for APP-y; $n = 51$ for APP-a). **c** Signal intensity of VGLUT1 levels (nTG-y vs. nTG-a: $P = 4.6 \times 10^{-6}$; nTG-y vs. APP-y: $P = 3.9\times 10^{-21}$; APP-y vs. APP-a: $P = 0.15$; nTG-a vs. APP-a: $P = 4.5 \times 10^{-21}$) in CA1 between groups (brain slice, $n = 79$ for nTG-y; $n = 80$ for nTG-a; $n = 70$ for APP-y; $n = 79$ for APP-a). nTG-y, non-transgenic young; nTG-a, non-transgenic adult; APP-y, APP young; APP-a, APP adult. Wilcoxon rank sum tests (two-sided) corrected for multiple comparisons using a Bonferroni-Holm correction were applied to analyze the data in Supplementary Fig. 23 **b-c**. Data in bar graphs are presented as mean values ± SEM; *$P < 0.05$, **$P < 0.01$, ***$P < 0.001$; n.s, not significant. Source data are provided as a Source Data file.

Supplementary Figure 24

Two-way ANOVA: VGLUT3 signal in the MEC

Source	Sum Square (10^4)	*df*	MS(10^4)	*F*	*p*
Age	11.34	1	11.34	35.83	= 1e-8
Genotype	19.34	1	19.34	61.09	= 3.4e-13
Interaction	2.36	1	2.36	7.46	= 0.0069
Error	61.42	194	0.32		
Total	92.71	197			

Two-way ANOVA: VGLUT1 signal in CA1

Source	Sum Square (10^4)	*df*	MS(10^4)	*F*	*p*
Age	15.28	1	15.28	9.94	= 0.0018
Genotype	631.66	1	631.66	410.95	= 2.1e-58
Interaction	38.18	1	38.18	24.84	= 1e-6
Error	467.27	304	1.54		
Total	1150.26	307			

Supplementary Figure 24. VGLUT3 levels in the MEC and VGLUT1 levels in the CA1 are both higher in APP mice. Two-way unbalanced ANOVAs compare the effects of age, genotype, and interaction on VGLUT3 and VGLUT1 signal levels in the MEC and CA1 respectively.

df = degrees of freedom, MS = mean square.
