# Supplementary material for: Disruption of the grid cell network in a mouse model of early Alzheimer’s disease
Source: Nat Commun. 2022 Feb 16;13:886. doi: 10.1038/s41467-022-28551-x (PMC8850598; doi:10.1038/s41467-022-28551-x)
Supplement: Supplementary file 3 — Reporting Summary [file 41467_2022_28551_MOESM3_ESM.pdf]

Corresponding author(s): Mark P. Brandon

Last updated by author(s): Dec 20, 2021

Reporting Summary

Nature Portfolio wishes to improve the reproducibility of the work that we publish. This form provides structure for consistency and transparency in reporting. For further information on Nature Portfolio policies, see our Editorial Policies and the Editorial Policy Checklist.

Statistics

For all statistical analyses, confirm that the following items are present in the figure legend, table legend, main text, or Methods section.

n/a	Confirmed	
☐	☒	The exact sample size (*n*) for each experimental group/condition, given as a discrete number and unit of measurement
☐	☒	A statement on whether measurements were taken from distinct samples or whether the same sample was measured repeatedly
☐	☒	The statistical test(s) used AND whether they are one- or two-sided<br>*Only common tests should be described solely by name; describe more complex techniques in the Methods section.*
☒	☐	A description of all covariates tested
☐	☒	A description of any assumptions or corrections, such as tests of normality and adjustment for multiple comparisons
☐	☒	A full description of the statistical parameters including central tendency (e.g. means) or other basic estimates (e.g. regression coefficient) AND variation (e.g. standard deviation) or associated estimates of uncertainty (e.g. confidence intervals)
☐	☒	For null hypothesis testing, the test statistic (e.g. F, t, r) with confidence intervals, effect sizes, degrees of freedom and P value noted<br>*Give P values as exact values whenever suitable.*
☒	☐	For Bayesian analysis, information on the choice of priors and Markov chain Monte Carlo settings
☒	☐	For hierarchical and complex designs, identification of the appropriate level for tests and full reporting of outcomes
☐	☒	Estimates of effect sizes (e.g. Cohen's *d*, Pearson's *r*), indicating how they were calculated

Our web collection on statistics for biologists contains articles on many of the points above.

Software and code

Policy information about availability of computer code

Data collection	Electrophysiological data was collected using Cheetah 5.0 software (Neuralynx, Inc).
Data analysis	Electrophysiological data was analyzed with Offline Sorter 2.8.8 (Plexon, Inc) and Matlab 2018b (Mathwork, Inc). Path integration behavioral data was analyzed with Matlab 2018b. Immunohistochemical data was analyzed with ImageJ 1.52d (NIH) and RStudio 2016 (RStudio, PBC). Immunoautoradiographic data was analyzed with MCID analysis software (InterFocus, Ltd).

For manuscripts utilizing custom algorithms or software that are central to the research but not yet described in published literature, software must be made available to editors and reviewers. We strongly encourage code deposition in a community repository (e.g. GitHub). See the Nature Portfolio guidelines for submitting code & software for further information.

Data

Policy information about availability of data

All manuscripts must include a data availability statement. This statement should provide the following information, where applicable:

- Accession codes, unique identifiers, or web links for publicly available datasets
- A description of any restrictions on data availability
- For clinical datasets or third party data, please ensure that the statement adheres to our policy

All data supporting the key findings of this study are available within the article, Supplementary Information and Source Data. Source data are provided with this paper.

All custom codes supporting the key findings of this study are available at the following GitHub page: https://github.com/johnson-ying/ying-et-al-2021, or via request to the corresponding author.

Field-specific reporting

Please select the one below that is the best fit for your research. If you are not sure, read the appropriate sections before making your selection.

☒ Life sciences ☐ Behavioural & social sciences ☐ Ecological, evolutionary & environmental sciences

For a reference copy of the document with all sections, see nature.com/documents/nr-reporting-summary-flat.pdf

Life sciences study design

All studies must disclose on these points even when the disclosure is negative.

Sample size	No statistical methods were used to predetermine sample size. Sample size was determined on the basis of prior reports on spatially tuned neurons in the MEC-hippocampal circuits of Alzheimer mouse models.<br><br>Cacucci, F., Yi, M., Wills, T. J., Chapman, P. & O'Keefe, J. Place cell firing correlates with memory deficits and amyloid plaque burden in Tg2576 Alzheimer mouse model. Proc. Natl. Acad. Sci. U. S. A. 105, 7863–7868 (2008).<br><br>Fu, H. et al. Tau pathology induces excitatory neuron loss, grid cell dysfunction, and spatial memory deficits reminiscent of early Alzheimer's disease. Neuron 93, 533-541.e5 (2017).
Data exclusions	Data requiring phase estimate of theta oscillation was excluded when phase could not be reliably estimated (the LFP signal showed >1% of signal saturation).
Replication	We used a large cohort of mice (38 transgenic and 30 non-transgenic) to replicate our findings. All attempts to replicate our findings were successful.
Randomization	Mice were allocated to experimental groups based purely on their genotype.
Blinding	Experimenters were blind to the genotype of the animal subjects for all surgeries and experiments that involved neural recordings or behavioral testing. Experimenters were not blind to immunohistochemical experiments because all brain tissue was processed the same way irregardless of animal genotype. Experimenters were not blind to immunoautoradiographic experiments because all brain tissue was processed the same way irregardless of animal genotype. Experimenters were blind to analysis prior to all statistical testing.

Reporting for specific materials, systems and methods

We require information from authors about some types of materials, experimental systems and methods used in many studies. Here, indicate whether each material, system or method listed is relevant to your study. If you are not sure if a list item applies to your research, read the appropriate section before selecting a response.

Materials & experimental systems

n/a	Involved in the study	
☐	☒	Antibodies
☒	☐	Eukaryotic cell lines
☒	☐	Palaeontology and archaeology
☐	☒	Animals and other organisms
☒	☐	Human research participants
☒	☐	Clinical data
☒	☐	Dual use research of concern

Methods

n/a	Involved in the study	
☒	☐	ChIP-seq
☒	☐	Flow cytometry
☒	☐	MRI-based neuroimaging

Antibodies

Antibodies used	- Mouse monoclonal anti-β-Amyloid, 1-16 Antibody (Biolegend, Cat# 803001, Clone: 6E10, Lot: B247600).<br>- BiotinylatedAnti-Mouse Ig Reagent, from Mouse on Mouse (M.O.M.®) Fluorescein Kit (Vector Laboratories, Cat#: FMK-2201).<br><br>- VGLUT1 (prepared in-house, originally used in:<br>Herzog E, Bellenchi GC, Gras C, et al. The existence of a second vesicular glutamate transporter specifies subpopulations of glutamatergic neurons. J Neurosci. 2001;21(22):RC181. doi:10.1523/JNEUROSCI.21-22-j0001.2001).<br>- VGLUT3 (Synaptic Systems, Cat# 135203)<br>- VGAT (Synaptic Systems, Cat# 131002)<br>- VAChT (Synaptic Systems, Cat# 139103)<br>- NR1 (Synaptic Systems, Cat# 114103)<br>- Goat anti-rabbit [125I]-IgG (PerkinElmer, Part# NEX155100UC)
Validation	Each lot of anti-β-Amyloid antibody is tested for quality control by the supplier using Western blot (https://www.biolegend.com/en-

Validation

us/products/purified-anti-beta-amyloid-1-16-antibody-11228).

We used brain sections of 18+ months old transgenic animals as positive controls and tested 3 concentrations (1 µg/ml, 2 µg/ml, 5 µg/ml) within the range recommended by the supplier (0,2 - 5 µg/ml), for immunohistochemistry applications.

VGLUT1 antibody is prepared in-house and was originally validated in Herzog et al. 2001.

VGLUT3, VGAT, VAChT and NR1 are tested for quality control by the supplier. Validation data are available on the manufacturer's website.
VGLUT3 - https://sysy.com/product/135203
VGAT - https://sysy.com/product/131002
VAChT - https://sysy.com/product/139103
NR1 - https://sysy.com/product/114103
Goat anti-rabbit - https://www.perkinelmer.com/product/goat-anti-rabbit-igg-whole-antibody-fr-nex155100uc

Animals and other organisms

Policy information about studies involving animals; ARRIVE guidelines recommended for reporting animal research

Laboratory animals

J20 APP male mice (B6.Cg-Zbtb20 Tg(PDGFB-APPSwInd) 20Lms/2Mmjax) were obtained from Jackson Laboratories (MMRRC stock #34836) and bred with female C57/BL6/j mice. Mice were individually housed on a 12-h light/dark cycle and underwent experiments during the light cycle. Housing room conditions of the mice were maintained at 20-22 degrees Celsius and 21-30% humidity.

Male and female offspring were used in this study, ranging in age from 3 to 7 months. Animals exceeding 18 months of age were only used in immunohistochemical experiments.

Wild animals

This study did not involve wild animals.

Field-collected samples

This study did not involve samples collected from the field.

Ethics oversight

All experiments were conducted in accordance with McGill University and Douglas Hospital Research Centre Animal Use and Care Committee (protocol #2015-7725) and in accordance with Canadian Institutes of Health Research guidelines.

Note that full information on the approval of the study protocol must also be provided in the manuscript.
